# Efficacy and safety of oxiracetam for cognitive and functional outcomes after acute traumatic brain injury in Chinese populations: a systematic review and meta-analysis of randomized controlled trials

**DOI:** 10.3389/fphar.2026.1894256

**Published:** 2026-09-21

**Authors:** Mimi Wang, Ning Cao, Zhangxuan Shou, Jueyi Pan

**Affiliations:** 1 Department of Pharmacy, The Second Affiliated Hospital of Zhejiang Chinese Medical University, Hangzhou, Zhejiang, China; 2 Department of Neurology, The Second Affiliated Hospital of Zhejiang Chinese Medical University, Hangzhou, Zhejiang, China

**Keywords:** activities of daily living, cognition, meta-analysis, neurological deficit, oxiracetam, safety, traumatic brain injury

## Abstract

**Background:**

Oxiracetam is used in China for cognitive dysfunction after traumatic brain injury (TBI), but its efficacy and safety remain uncertain. We assessed oxiracetam in adults with acute TBI.

**Methods:**

We searched six bibliographic databases and trial registries from inception through 28 February 2026, for randomized trials. Risk of bias was assessed with the Cochrane risk-of-bias tool (RoB 1), and evidence certainty with GRADE. We performed comparator-stratified random-effects meta-analyses and verified treatment regimens, assessment times, and numerical data against source reports.

**Results:**

Thirty-three randomized controlled trials involving 2,864 participants contributed to the quantitative synthesis. When both groups received the same or otherwise balanced background therapy, mean post-treatment MMSE scores were 4.67 points higher with oxiracetam (10 studies, n = 798; MD 4.67, 95% CI 3.07–6.28; I^2^ = 92%), and NIHSS scores were 3.22 points lower (8 studies, n = 685; MD −3.22, 95% CI −4.14 to −2.30; I^2^ = 58%). Compared with piracetam, mean post-treatment MMSE scores were 4.05 points higher with oxiracetam (8 studies, n = 739; MD 4.05, 95% CI 3.51–4.60; I^2^ = 0%). These MDs were between-group differences in endpoint scores, not within-patient changes. Barthel Index and GCS scores were also higher with oxiracetam in balanced-background comparisons, but heterogeneity was substantial. More participants met study-defined response criteria with oxiracetam than with control (5 studies, n = 558; RR 1.26, 95% CI 1.16–1.37; I^2^ = 0%). In balanced-background comparisons, reported adverse events or adverse reactions did not differ significantly between groups (5 studies, n = 504; RR 1.14, 95% CI 0.64–2.04; I^2^ = 0%), although reporting was sparse and incomplete.

**Conclusion:**

Oxiracetam was associated with higher short-term MMSE scores and, in some comparisons, lower NIHSS scores and higher Barthel Index and GCS scores after acute TBI. Risk of bias, clinical heterogeneity, variable assessment timing, incomplete safety reporting, and low or very low certainty of evidence preclude firm conclusions about durable benefit or routine clinical use.

**Systematic Review Registration:**

https://www.crd.york.ac.uk/prospero/display_record.php?ID=CRD420261394733, identifier CRD420261394733.

## 1 Introduction

Traumatic brain injury (TBI) is a major cause of death and long-term disability worldwide. Survivors may experience persistent cognitive, neurological, behavioral, and functional impairments that limit independence, social participation, and quality of life (Blennow et al., 2016; Dewan et al., 2019; Maas et al., 2017; Maas et al., 2022). Cognitive and functional recovery are therefore important treatment goals.

Oxiracetam is a hydroxylated pyrrolidone derivative structurally related to piracetam. Experimental work suggests effects on cholinergic and glutamatergic neurotransmission, synaptic plasticity, neuronal metabolism, and inflammatory responses (Gouliaev and Senning, 1994; Youn et al., 2023). These findings provide a biological rationale but do not establish clinical efficacy.

Relevant Chinese and international guidance documents differ in clinical phase, target population, and scope. The 2017 Chinese consensus on early rehabilitation after severe TBI emphasizes early multidisciplinary rehabilitation, cognitive screening with the MMSE or MoCA, and training directed at attention, memory, and executive function; a subsequent Chinese neurorestorative guideline cautions that pharmacotherapy alone should not be the principal restorative strategy. International guidance addresses partly different phases and injury severities: INCOG 2.0 prioritizes individualized, goal-oriented cognitive rehabilitation, whereas the U.S. Department of Veterans Affairs/Department of Defense guideline recommends a time-limited trial of clinician-directed cognitive rehabilitation for persistent cognitive symptoms after mild TBI. None of these documents provides an oxiracetam-specific recommendation (Chinese Medical Association Neurosurgery Branch and Chinese Neurosurgical Critical Care Collaboration Group, 2017; Yang et al., 2022; Bayley et al., 2023; U.S. Department of Veterans Affairs and Department of Defense, 2021). The contrast between the Chinese clinical-trial literature on oxiracetam and the absence of an oxiracetam-specific recommendation in these guidance documents provides the rationale for a TBI-specific systematic appraisal.

Most randomized evidence on oxiracetam after TBI comes from small trials conducted in China. These trials address three distinct questions: whether oxiracetam adds benefit to usual care, whether it is more effective than piracetam, and whether it adds benefit to a nimodipine-based regimen. Comparability is further limited by differences in formulation, route, dose, treatment duration, time from injury to treatment, outcome assessment, injury severity, and background care. Consequently, estimates from short-course intravenous racemic oxiracetam, oral regimens, and 90-day evaluations of L-oxiracetam may not reflect the same clinical effect.

Accordingly, we systematically reviewed randomized trials conducted in China that evaluated oxiracetam in adults with acute TBI. We estimated efficacy and safety separately by comparator and outcome domain, verified treatment regimens and numerical data against the source reports, and summarized the contemporary phase 3 LOCATE trial narratively because its intervention and follow-up differed from those of the earlier trials.

## 2 Materials and methods

### 2.1 Protocol, registration, and reporting

This review was reported in accordance with the PRISMA 2020 statement (Page et al., 2021) and registered in PROSPERO (CRD420261394733). Registration was completed after the search cutoff but before the final quantitative synthesis. The review question, eligibility criteria, outcome domains, comparator strata, and statistical approach were specified before the final analyses.

### 2.2 Study design and PICOS framework

Eligibility was defined using the PICOS framework. The population comprised adults with acute TBI in China; a formal diagnosis of post-TBI cognitive disorder was not required. Eligible interventions were oral or intravenous racemic oxiracetam or L-oxiracetam, administered alone or as the only additional active intervention. Comparators were placebo or the same or otherwise balanced background care, piracetam administered with comparable background care, or nimodipine alone when both groups received nimodipine. Outcomes included cognitive function, activities of daily living, neurological impairment, level of consciousness or global recovery, investigator-defined response, and safety. Parallel-group randomized controlled trials with extractable arm-level data were eligible. Because the comparator groups addressed distinct clinical questions, they were analyzed separately.

### 2.3 Inclusion and exclusion criteria

Studies were eligible if they enrolled adults with acute TBI (including acute craniocerebral injury, craniocerebral trauma, or cerebral contusion and laceration, as described in the source report); randomly assigned participants to an eligible oxiracetam regimen or comparator; and reported at least one prespecified efficacy or safety outcome with extractable group-level data. The primary outcome was cognitive function measured with a named instrument, with the Mini-Mental State Examination (MMSE) prioritized. Secondary outcomes were the Barthel Index (BI), National Institutes of Health Stroke Scale (NIHSS), Glasgow Coma Scale (GCS), Glasgow Outcome Scale or Extended Glasgow Outcome Scale, investigator-defined response, and safety. No language restrictions were applied.

We excluded nonrandomized and quasi-randomized studies, observational studies, animal or *in vitro* studies, reviews, protocols, conference abstracts without full reports, and mixed populations from which TBI data could not be separated. Reports were also excluded when both groups received oxiracetam, when co-interventions other than oxiracetam were clearly imbalanced, or when key data were not extractable. For unresolved problems confined to a single outcome, the affected outcome was excluded while other usable data from the study were retained.

### 2.4 Information sources and search strategy

PubMed, Cochrane CENTRAL, China National Knowledge Infrastructure, Wanfang, VIP, and SinoMed/Chinese Biomedical Literature Database were searched from inception to 28 February 2026. We also searched ClinicalTrials.gov, the World Health Organization International Clinical Trials Registry Platform, the Chinese Clinical Trial Registry, and the Chinese Drug Clinical Trial Registration and Information Disclosure Platform. Search strategies combined English and Chinese terms for oxiracetam and TBI without outcome terms or randomized-trial filters. Reference lists of included studies and relevant reviews were screened. Full strategies are provided in Supplementary Table S1.

### 2.5 Study selection and data extraction

Records were imported into EndNote 21. Software-assisted deduplication was followed by manual verification. Two reviewers independently screened titles and abstracts and assessed potentially eligible full texts. Disagreements were resolved by discussion or, when necessary, adjudication by a third reviewer.

Two reviewers independently extracted participant characteristics, TBI severity, intervention and comparator regimens, background care and co-interventions, formulation, dose, frequency, route, treatment duration, time from injury to treatment initiation, outcome definitions, assessment times, and numerical data required for effect estimation. For each continuous outcome, we recorded the sample size in each group, instrument name, baseline value when reported, post-treatment or follow-up value, measure of dispersion, assessment time, and statistical notation used in the source report. We also extracted information required for the risk-of-bias assessment. Discrepancies were resolved by consensus after verification against the source article or registry record.

### 2.6 Treatment regimens, outcome timing, and data verification

We summarized study-level differences in oxiracetam formulation, dose, route, treatment duration, and outcome assessment time before interpreting the pooled estimates. Formal subgroup analyses by dose, route, or duration were not performed because too few studies were available within clinically coherent strata, comparator and outcome definitions differed across strata, and treatment-initiation or assessment times were often incompletely reported. We therefore classified regimens into clinically meaningful groups and considered these differences descriptively when interpreting heterogeneity.

All extracted numerical data were checked against the source reports. We distinguished baseline values, post-treatment endpoint values, and change scores; recorded whether variability was reported as an SD, SE, CI, or another statistic; and did not combine endpoint values with change scores in the same analysis. Values that could not be verified, were internally inconsistent, or were incompatible with the instrument range were excluded from the relevant quantitative analysis rather than corrected or imputed.

Barthel Index outcomes were analyzed using mean differences only when the source report explicitly identified the instrument as the Barthel Index/BI, used a 0–100 scale, and scored higher values as better function. All five reports met these criteria, although none reported the exact version or item wording. No differently named activities-of-daily-living instrument was included in an unstandardized mean-difference analysis. Study-level regimens, extracted data, and Barthel Index measurement characteristics are reported in Supplementary Tables S5–S7.

### 2.7 Risk-of-bias assessment

Risk of bias was assessed using the original Cochrane risk-of-bias tool for randomized trials (RoB 1) (Higgins et al., 2011). The assessed domains were random-sequence generation, allocation concealment, blinding of participants and personnel, blinding of outcome assessment, incomplete outcome data, selective reporting, and other bias. Each domain was rated as low, unclear, or high risk. Random-sequence generation was rated as low risk only when an adequate method, such as a random-number table or computer-generated sequence, was reported. The other-bias domain captured important baseline or co-intervention imbalances, possible duplicate reporting, and unresolved data inconsistencies. LOCATE was assessed using the same domains, with particular attention to allocation concealment, blinding, prespecified reporting, and attrition through day 90.

### 2.8 Certainty of evidence

Certainty of evidence was assessed for each comparator and outcome using GRADE (Guyatt et al., 2008). We considered risk of bias, inconsistency, indirectness, imprecision, and publication bias and rated certainty as high, moderate, low, or very low. For safety outcomes, we also considered whether adverse events were monitored prospectively, whether serious and treatment-related events were distinguished, and whether arm-specific event counts were reported.

### 2.9 Statistical analysis

Meta-analyses were performed in Review Manager 5.4.1 (Cochrane Collaboration, 2020). Continuous outcomes measured with the same instrument were summarized as mean differences (MDs) with 95% confidence intervals (CIs) using the inverse-variance method. Barthel Index outcomes were analyzed as MDs only when the source explicitly identified the instrument as the Barthel Index/BI, used a 0–100 scale, and scored higher values as better function. Dichotomous outcomes were summarized as risk ratios (RRs) with 95% CIs using the Mantel–Haenszel method. Random-effects models were used because clinical and methodological heterogeneity was expected. Between-study variance was estimated using the DerSimonian–Laird method (DerSimonian and Laird, 1986).

Analyses were stratified into three comparator groups: oxiracetam added to the same or otherwise balanced background therapy versus control; oxiracetam versus piracetam with comparable background care; and oxiracetam plus nimodipine versus nimodipine alone. Investigator-defined response was treated as a supplementary outcome because definitions varied across reports. Statistical heterogeneity was quantified using I^2^ and τ^2^ and interpreted together with clinical differences in formulation, route, treatment duration, comparator, and assessment time. Sensitivity analyses examined uncertain scale classifications. Implausible, internally inconsistent, or unverifiable values were excluded rather than corrected or imputed.

## 3 Results

The searches identified 734 records, including 717 from bibliographic databases and 17 from trial registries. After 287 duplicates had been removed, 447 records underwent title and abstract screening, of which 346 were excluded. Full-text reports were sought for 101 records, but two could not be retrieved. Of the 99 reports assessed for eligibility, 65 were excluded for the reasons listed in Supplementary Table S2. Thirty-four randomized trial reports met the inclusion criteria: 33 reports involving 2,864 participants contributed to the quantitative synthesis, whereas the 590-participant LOCATE phase 3 trial was included in the narrative synthesis. The study-selection process is shown in Figure 1.

**FIGURE 1:**
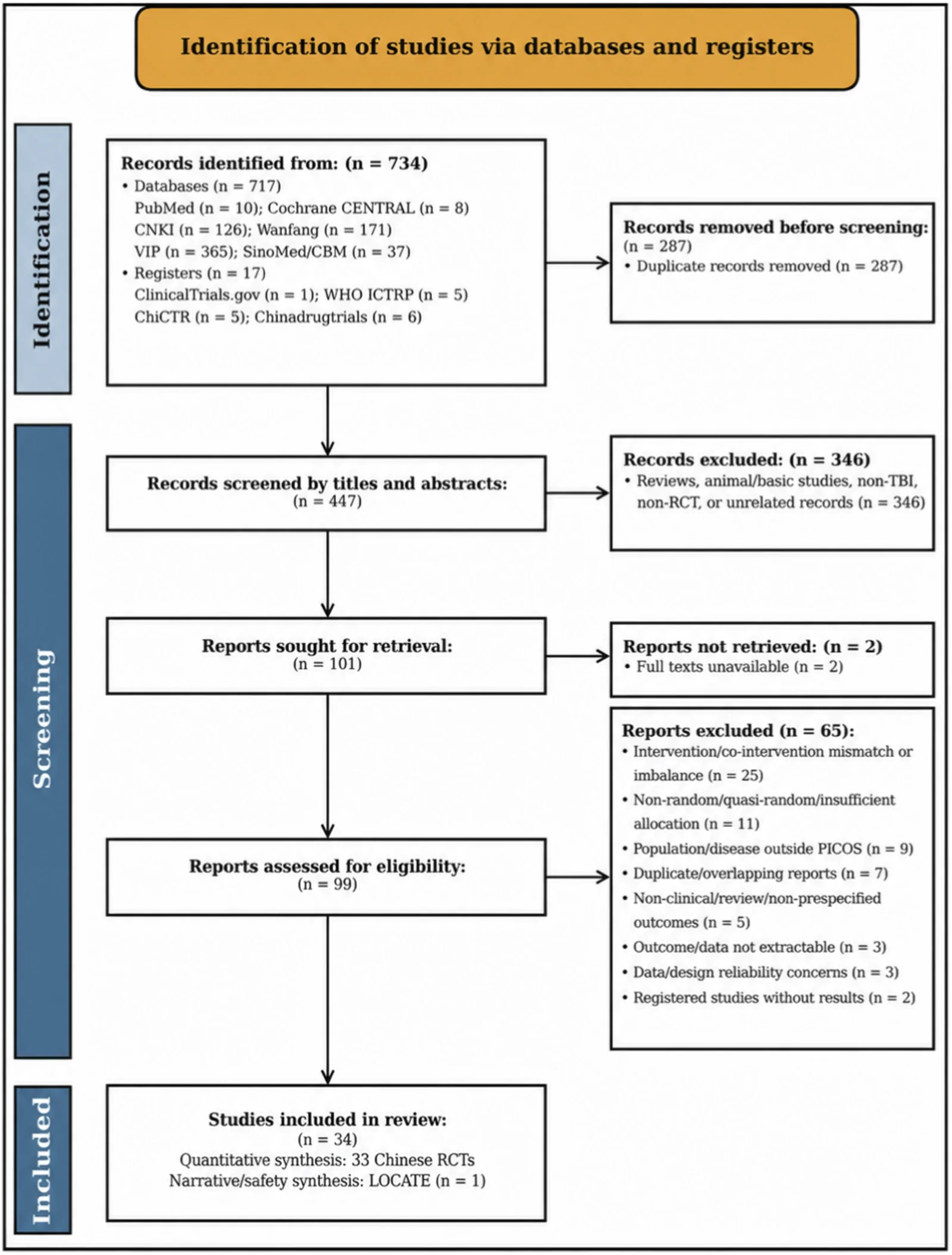
PRISMA 2020 flow diagram of study selection.

### 3.1 Meta-analysis

#### 3.1.1 Study characteristics and risk of bias

The 33 randomized trial reports from China were published from 2005 to 2022 and included 2,864 participants; individual sample sizes ranged from 35 to 150. Eighteen reports evaluated oxiracetam added to the same or otherwise balanced background therapy, 10 compared oxiracetam with piracetam under comparable background care, and five compared oxiracetam plus nimodipine with nimodipine alone. Twenty-nine reports used intravenous racemic oxiracetam and four used oral capsules (Table 1). Twenty-seven reports used short-course intravenous regimens (≤21 days), including one in which outcomes were assessed during longer-term follow-up. The remaining six reports comprised four oral regimens, one 12-week intravenous regimen, and one intravenous regimen for which treatment duration and assessment timing were not reported. Among studies reporting treatment duration, courses ranged from 12 to 180 days. Outcomes were usually assessed at the end of treatment or during early follow-up, although several reports used longer follow-up. Study-level details are provided in Supplementary Table S5.

Outcome timing and measurement varied across reports. Several studies provided only post-treatment values, whereas others reported serial GCS or NIHSS assessments at multiple early time points. For studies with multiple assessments, the endpoint included in each quantitative synthesis and its exact timing were documented to permit evaluation of time-point heterogeneity. Study-level continuous extraction details, including baseline and endpoint values, group-specific sample sizes, source notation, and source page or table, are provided in Supplementary Table S6A; event data are provided in Supplementary Table S6B. Barthel Index characteristics are summarized in Supplementary Table S7.

Random-sequence generation was rated at low risk in 13 reports and at unclear risk in 20. Allocation concealment was adequately reported in one study and was unclear in the remaining 32. Blinding of participants, personnel, and outcome assessors was generally not described. Most reports were rated at low risk for incomplete outcome data, but selective reporting was rated as unclear because protocols or registrations were usually unavailable. Five reports were rated at high risk for other bias because of data concerns or co-intervention imbalance. Supplementary Figure S1 presents the domain-level graph and study-level judgments.

#### 3.1.2 Cognitive outcome: MMSE

Among trials in which both groups received the same or otherwise balanced background therapy, mean post-treatment MMSE scores were 4.67 points higher with oxiracetam than with the control regimen (10 studies, n = 798; MD 4.67, 95% CI 3.07–6.28; I^2^ = 92%). Compared with piracetam, oxiracetam was associated with mean post-treatment MMSE scores that were 4.05 points higher (8 studies, n = 739; MD 4.05, 95% CI 3.51–4.60; I^2^ = 0%). These results are presented in Table 2; Figure 2.

**TABLE 1:** Evidence composition by comparator.

Evidence component	Reports	Participants	Role in synthesis	Main outcomes
Oxiracetam add-on or balanced background therapy vs. control	18	1,520	Primary quantitative comparison	MMSE; Barthel Index (BI); NIHSS; GCS; investigator-defined response; safety
Oxiracetam vs. piracetam	10	879	Active-comparator quantitative comparison	MMSE; Barthel Index (BI); NIHSS; GCS; investigator-defined response; safety
Oxiracetam + nimodipine vs. nimodipine	5	465	Separate quantitative comparison	NIHSS; GCS; investigator-defined response; safety
LOCATE phase 3 trial	1	590	Separate narrative efficacy and structured safety synthesis	LOTCA; MMSE; MoCA; ADL; GCS; GOSE; adverse events; serious adverse events; discontinuation; mortality

**TABLE 2:** Meta-analysis results by comparator.

Comparison	Outcome	Studies	Participants	Effect	95% CI	I^2^ (%)	Certainty
Add-on/balanced background	MMSE (primary)	10	798	MD 4.67	3.07–6.28	92	Very low
Add-on/balanced background	MMSE (sensitivity)a	11	870	MD 4.69	3.29–6.09	91	Not rated
Add-on/balanced background	Barthel Index (BI)	4	378	MD 12.79	7.09–18.49	81	Very low
Add-on/balanced background	NIHSS (primary)	8	685	MD −3.22	−4.14 to −2.30	58	Low
Add-on/balanced background	NIHSS (sensitivity)a	9	757	MD −3.37	−4.20 to −2.53	61	Not rated
Add-on/balanced background	GCS (primary)	9	783	MD 2.64	1.94–3.34	96	Very low
Add-on/balanced background	Investigator-defined response	5	558	RR 1.26	1.16–1.37	0	Very low
Add-on/balanced background	Reported adverse events/adverse reactions	5	504	RR 1.14	0.64–2.04	0	Very low
Oxiracetam vs. piracetam	MMSE	8	739	MD 4.05	3.51–4.60	0	Low
Oxiracetam vs. piracetam	Barthel Index (BI)	1	50	MD 21.18	15.02–27.34	NA	Very low
Oxiracetam vs. piracetam	NIHSS	9	759	MD −3.71	−4.54 to −2.87	64	Low
Oxiracetam vs. piracetam	GCS	8	775	MD 2.08	1.22–2.94	90	Very low
Oxiracetam vs. piracetam	Investigator-defined response	1	120	RR 1.16	0.96–1.41	NA	Very low
Oxiracetam vs. piracetam	Reported adverse reactions	1	95	RR 0.16	0.02–1.30	NA	Very low
Oxiracetam + nimodipine vs. nimodipine	NIHSS	4	365	MD −4.01	−4.52 to −3.49	0	Low
Oxiracetam + nimodipine vs. nimodipine	GCS	2	230	MD 4.64	−2.27 to 11.55	100	Very low
Oxiracetam + nimodipine vs. nimodipine	Investigator-defined response	4	405	RR 1.29	1.17–1.42	0	Very low

Only primary analyses informed GRADE. a, Bai (2015) unnamed scales treated as MMSE, or NIHSS, in sensitivity analyses only. Unverifiable, internally inconsistent, or implausible values were excluded rather than corrected or imputed. NA, not applicable.

**FIGURE 2:**
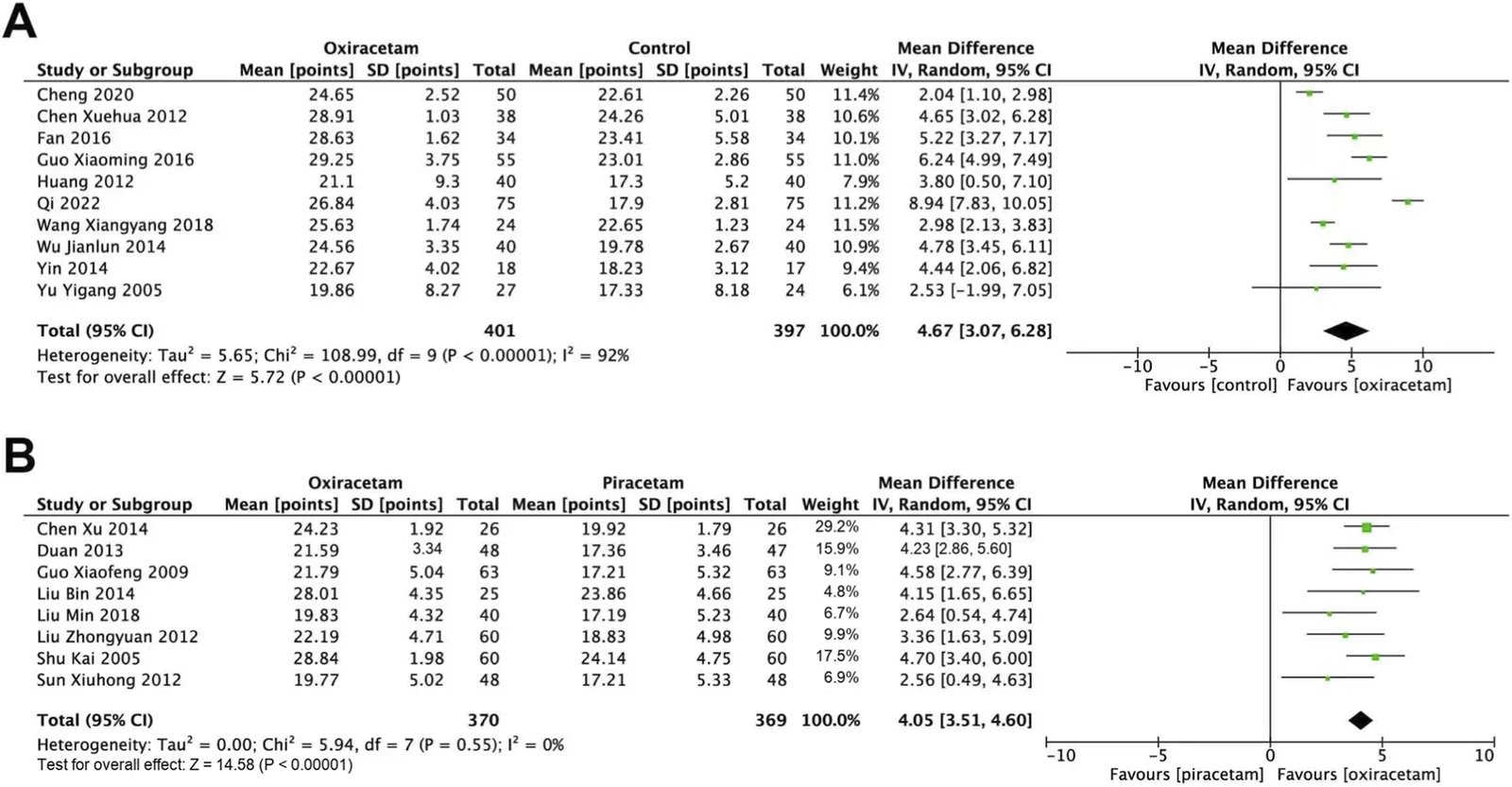
Forest plots of post-treatment MMSE scores in the primary analyses. **(A)** Oxiracetam added to the same or balanced background therapy versus control: 10 studies, n = 798, MD 4.67 (95% CI 3.07–6.28), I^2^ = 92%. **(B)** Oxiracetam versus piracetam: 8 studies, n = 739, MD 4.05 (95% CI 3.51–4.60), I^2^ = 0%; the source-reported endpoint SD of 3.34 was used for Duan (2013). Analyses used inverse-variance random-effects models in Review Manager 5.4.1 and were not pooled across comparator strata.

#### 3.1.3 Other efficacy outcomes

##### 3.1.3.1 Barthel index

Among trials with the same or otherwise balanced background therapy, mean post-treatment Barthel Index (BI) scores were 12.79 points higher with oxiracetam than with control (4 studies, n = 378; MD 12.79, 95% CI 7.09–18.49; I^2^ = 81%). One piracetam-controlled trial also reported a higher mean BI score with oxiracetam (n = 50; MD 21.18, 95% CI 15.02–27.34). All five reports explicitly identified the instrument as the Barthel Index/BI and used a 0–100 scale on which higher scores indicate better function; however, the exact version and item wording were not reported. The pooled MD should therefore be interpreted as a summary difference in the reported BI scores, not as a uniform 12.79-point improvement in functional independence. Results are shown in Table 2; Supplementary Figure S2, and instrument details are provided in Supplementary Table S7.

##### 3.1.3.2 Neurological deficit

Mean post-treatment NIHSS scores were lower with oxiracetam than with control when both groups received the same or otherwise balanced background therapy (8 studies, n = 685; MD −3.22, 95% CI −4.14 to −2.30; I^2^ = 58%) and lower with oxiracetam than with piracetam (9 studies, n = 759; MD −3.71, 95% CI −4.54 to −2.87; I^2^ = 64%). Mean NIHSS scores were also lower with oxiracetam plus nimodipine than with nimodipine alone (4 studies, n = 365; MD −4.01, 95% CI −4.52 to −3.49; I^2^ = 0%). Lower scores indicate less neurological deficit. Results are shown in Table 2; Supplementary Figure S3.

##### 3.1.3.3 Level of consciousness and investigator-defined response

Mean post-treatment GCS scores were higher with oxiracetam than with control when both groups received the same or otherwise balanced background therapy (9 studies, n = 783; MD 2.64, 95% CI 1.94–3.34; I^2^ = 96%) and higher with oxiracetam than with piracetam (8 studies, n = 775; MD 2.08, 95% CI 1.22–2.94; I^2^ = 90%). The estimate for oxiracetam plus nimodipine versus nimodipine alone was imprecise (2 studies, n = 230; MD 4.64, 95% CI −2.27 to 11.55; I^2^ = 100%). Zhai Z. et al. (2015) was excluded from the primary add-on analysis because the reported day-21 control-group dispersion was incompatible with the range of the GCS; no decimal-place correction was made. The proportion of participants meeting the response criteria defined by the individual studies was higher with oxiracetam than with control in the balanced-background comparison (5 studies, n = 558; RR 1.26, 95% CI 1.16–1.37; I^2^ = 0%) and higher with oxiracetam plus nimodipine than with nimodipine alone (4 studies, n = 405; RR 1.29, 95% CI 1.17–1.42; I^2^ = 0%). Liu and Zhang (2020) was excluded from the latter analysis because the reported control-group percentage and category counts implied different numbers of responders. The only piracetam-controlled trial showed no statistically significant between-group difference (n = 120; RR 1.16, 95% CI 0.96–1.41). Because response definitions varied across trials, this outcome should be interpreted cautiously. Results are shown in Table 2; Supplementary Figures S4,S5.

#### 3.1.4 Safety outcomes

Safety outcomes were incompletely and inconsistently reported across the 33 trials conducted in China. Among the five trials with verifiable arm-specific counts, the pooled risk of reported adverse events or adverse reactions did not differ statistically between oxiracetam and control when background therapy was the same or otherwise balanced (n = 504; RR 1.14, 95% CI 0.64–2.04; I^2^ = 0%). Reports without a control-arm event count were excluded from the quantitative synthesis. The estimate from one piracetam-controlled trial was highly imprecise (n = 95; RR 0.16, 95% CI 0.02–1.30). Three additional reports stated that no adverse reactions occurred in either group and were summarized narratively. Results are presented in Table 3; Figure 3.

**TABLE 3:** Structured synthesis of safety evidence.

Evidence source	Safety outcome	Data	Effect/synthesis	Interpretation
Chinese RCTs: Add-on/balanced background—primary	Reported adverse events/adverse reactions	5 studies, n = 504	RR 1.14 (95% CI 0.64–2.04)	No statistically significant difference; source definitions and surveillance methods varied
Chinese RCTs: oxiracetam vs. piracetam	Reported adverse reactions	1 study, n = 95	RR 0.16 (95% CI 0.02–1.30)	Very imprecise; insufficient for inference
Chinese RCTs: Double-zero reports	Reported adverse reactions	3 studies, n = 322	Not estimable	Both groups reported zero adverse reactions; summarized narratively
Chinese RCTs: All-cause mortality	Follow-up mortality	2 studies, n = 176	Not pooled	Deaths were not adjudicated as treatment related, and follow-up differed
LOCATE	Any adverse event	155/235; 157/236; 80/119	Structured narrative synthesis	L-oxiracetam; racemic oxiracetam; placebo, respectively
LOCATE	Serious adverse event	13/235; 18/236; 15/119	Structured narrative synthesis	No treatment-related serious adverse event was reported
LOCATE	Permanent discontinuation because of adverse events	1/235; 9/236; 7/119	Structured narrative synthesis	Reported separately by treatment group
LOCATE	Treatment-related adverse event	22/235; 41/236; 11/119	Overall P = 0.049	The proportion was highest with racemic oxiracetam

**FIGURE 3:**
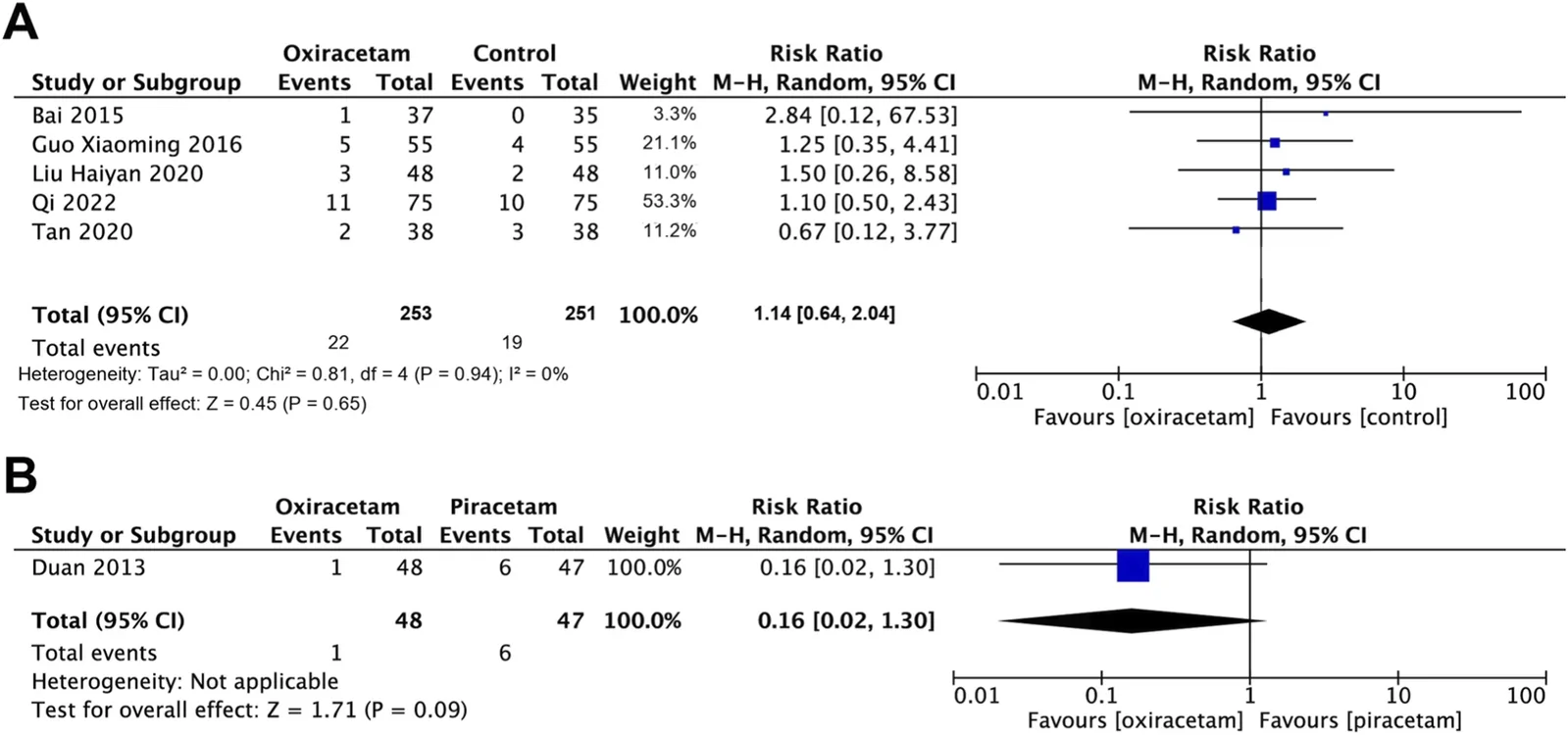
Forest plots of reported adverse events or adverse reactions in the primary analyses. **(A)** Oxiracetam added to the same or balanced background therapy versus control: 5 studies, n = 504, RR 1.14 (95% CI 0.64–2.04), I^2^ = 0%. **(B)** Oxiracetam versus piracetam: 1 study, n = 95, RR 0.16 (95% CI 0.02–1.30). Analyses used Mantel–Haenszel random-effects models in Review Manager 5.4.1; reports with missing arm-specific counts and double-zero reports were summarized narratively.

#### 3.1.5 Sensitivity analyses and small-study effects

Sensitivity analyses examined the classification of unnamed outcome scales. The primary analysis did not classify the unnamed mental-status and neurological-deficit scales in Bai (2015) as MMSE and NIHSS, respectively. Treating those unnamed scales as MMSE and NIHSS did not alter the direction of effect (MMSE: 11 studies, n = 870; MD 4.69, 95% CI 3.29–6.09; NIHSS: 9 studies, n = 757; MD −3.37, 95% CI −4.20 to −2.53). Source verification also identified an unreported control-group safety count in Wu (2014) and an internally inconsistent control-group response count in Liu and Zhang (2020); these data were excluded from the relevant quantitative syntheses. The GCS data from Zhai Z. et al. (2015) were excluded from the primary synthesis because the source table reported a dispersion value incompatible with the scale range. Source values and analysis decisions are detailed in Supplementary Tables S6A,6B.

Figure 4 presents the contour-enhanced funnel plot for the primary MMSE analysis in the balanced-background comparison (k = 10). The sparse and asymmetric distribution may reflect publication bias, clinical heterogeneity, chance, or other small-study effects. Given the small number of studies and substantial heterogeneity, the plot is exploratory and cannot establish publication bias.

**FIGURE 4:**
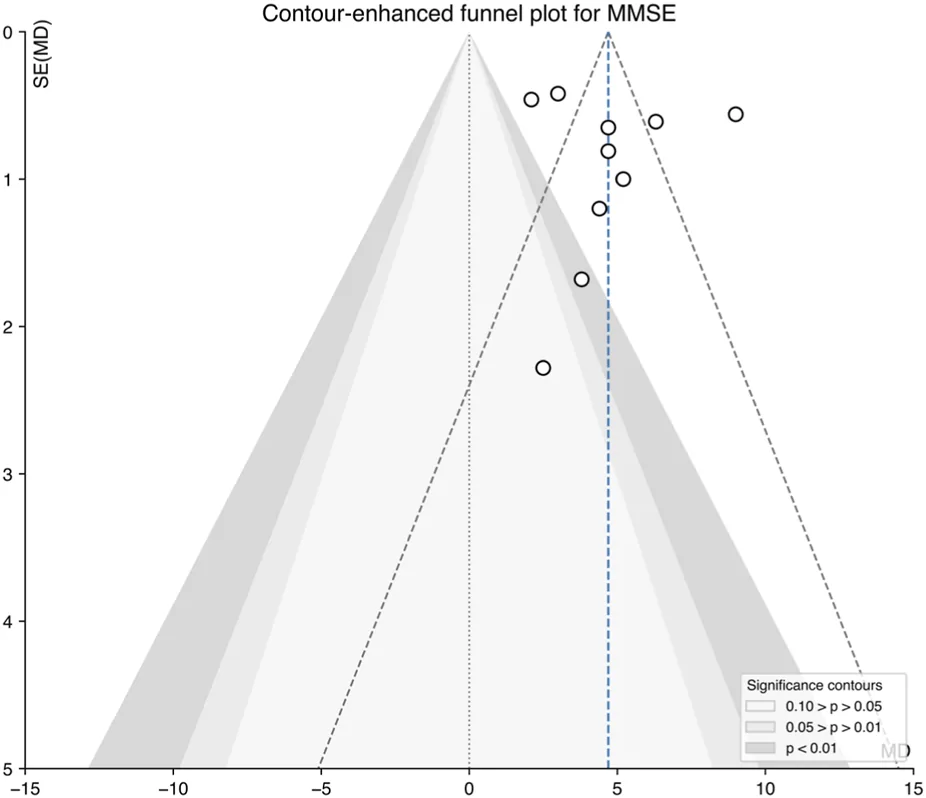
Contour-enhanced funnel plot for the primary MMSE analysis in the add-on or balanced-background comparison (k = 10; pooled MD 4.67). The vertical gray dotted line denotes the null effect, the vertical blue dashed line the random-effects pooled estimate, the oblique gray dashed lines the pseudo 95% limits, and the shaded regions two-sided significance contours relative to the null. Because only 10 studies were available and heterogeneity was substantial, the plot is exploratory and cannot establish publication bias.

#### 3.1.6 Certainty of evidence

Certainty of the evidence from the 33 randomized trials conducted in China was generally low or very low. Downgrading reflected risk of bias, inconsistency in continuous outcomes, indirectness arising from outcome choice or shared treatment background, imprecision, incomplete harm reporting, and possible small-study effects. Certainty was low for MMSE and NIHSS in the piracetam-controlled comparison and for NIHSS in the nimodipine-background comparison; most other outcomes were rated very low. Table 4 summarizes the GRADE judgments, and detailed comparison-specific summary-of-findings results are provided in Supplementary Table S4.

**TABLE 4:** GRADE certainty-of-evidence summary.

Comparison/source	Outcome	Certainty	Main reasons for rating
Add-on/balanced background—primary	MMSE	Very low	Risk of bias; substantial inconsistency; indirectness; possible small-study effects
Add-on/balanced background	Barthel Index (BI)	Very low	Risk of bias; substantial inconsistency; indirectness
Add-on/balanced background—primary	NIHSS	Low	Risk of bias; inconsistency
Add-on/balanced background—primary	GCS	Very low	Risk of bias; high heterogeneity; indirectness
Oxiracetam vs. piracetam	MMSE	Low	Risk of bias and study limitations despite low statistical heterogeneity
Oxiracetam vs. piracetam	NIHSS	Low	Risk of bias; heterogeneity
Oxiracetam vs. piracetam	GCS	Very low	Risk of bias; high heterogeneity
Oxiracetam + nimodipine vs. nimodipine	NIHSS	Low	Risk of bias; indirectness of the shared treatment background
Chinese RCT safety evidence—primary	Reported adverse events/adverse reactions	Very low	Incomplete, inconsistent, and sparse harm reporting
LOCATE	Day-90 LOTCA	Moderate	Downgraded mainly for attrition and missing-data considerations
LOCATE	Structured safety outcomes	Moderate	Single trial; limited precision for rare events

GRADE, ratings apply to the primary analyses. Values that could not be verified from the source report were excluded from the relevant quantitative synthesis.

### 3.2 Systematic review

LOCATE was the only eligible trial that was not combined quantitatively with the earlier randomized trials. This multicenter, double-blind, phase 3 trial enrolled 590 participants at 51 hospitals in China and randomly assigned them to L-oxiracetam, racemic oxiracetam, or placebo, with efficacy and safety outcomes assessed through day 90. More than 90% of the participants had mild TBI. LOCATE was summarized narratively because it included an L-oxiracetam arm, used a different primary cognitive instrument, followed participants for 90 days, and estimated adjusted change from baseline rather than unadjusted endpoint scores.

Centralized randomization, allocation concealment, double blinding, and prespecified analyses supported low-risk judgments for the corresponding bias domains. Attrition remained a concern because 146 of the 590 randomized participants withdrew before day 90, although the reported intention-to-treat and missing-data sensitivity analyses produced broadly consistent findings. Selective reporting was judged to be at low risk on the basis of the registered protocol and statistical analysis plan.

At day 90, the adjusted least-squares mean changes in Loewenstein Occupational Therapy Cognitive Assessment scores were 20.45 (95% CI 17.23–23.66) with L-oxiracetam, 15.90 (95% CI 12.71–19.10) with racemic oxiracetam, and 11.47 (95% CI 7.75–15.20) with placebo. The adjusted difference for L-oxiracetam was 8.97 points (95% CI 5.69–12.26) relative to placebo and 4.54 points (95% CI 1.85–7.23) relative to racemic oxiracetam. In contrast, the adjusted day-90 MMSE differences for L-oxiracetam were smaller, and their confidence intervals included zero: 0.71 points (95% CI −0.16 to 1.57) relative to placebo and 0.48 points (95% CI −0.23 to 1.18) relative to racemic oxiracetam (Liu et al., 2025).

Any adverse event was reported in 155 of 235 participants receiving L-oxiracetam, 157 of 236 receiving racemic oxiracetam, and 80 of 119 receiving placebo. Serious adverse events occurred in 13 of 235, 18 of 236, and 15 of 119 participants, respectively; none was considered treatment related. Treatment-related adverse events occurred in 22 of 235, 41 of 236, and 11 of 119 participants, respectively (overall P = 0.049), and were most frequent in the racemic-oxiracetam group. Permanent discontinuation because of adverse events occurred in 1 of 235, 9 of 236, and 7 of 119 participants, respectively (Liu et al., 2025).

## 4 Discussion

Across comparator-specific meta-analyses, oxiracetam was associated with higher short-term MMSE scores both when added to the same or otherwise balanced background therapy and when compared directly with piracetam. Some comparisons also showed higher Barthel Index and GCS scores, lower NIHSS scores, or higher investigator-defined response rates with oxiracetam. However, substantial heterogeneity and generally low or very low certainty of evidence preclude conclusions about sustained cognitive recovery or durable improvement in daily functioning. Importantly, the reported MDs are between-group differences in endpoint scores, not estimates of improvement within individual participants.

Comparator choice and outcome selection are central to interpretation. Add-on trials estimate the incremental effect of oxiracetam when background treatment is shared or balanced, whereas trials comparing oxiracetam with piracetam estimate relative efficacy between two nootropic agents and cannot establish efficacy relative to placebo or usual care. The instruments also measure different domains: the MMSE is a global cognitive screening tool; the Barthel Index assesses basic activities of daily living; the NIHSS measures neurological deficit but was developed for stroke; and the GCS reflects level of consciousness rather than cognition. Recovery after TBI encompasses cognition, behavior, neurological function, independence, participation, and quality of life, and favorable findings across these scales should not be treated as a single uniform effect on cognitive recovery (Blennow et al., 2016; Maas et al., 2017; Maas et al., 2022).

The closest previous quantitative synthesis was Wang et al. (2016), which applied a broad cerebral-organic-syndrome eligibility framework and included nine randomized trials (n = 1,347) comparing oxiracetam with piracetam. It reported an MMSE weighted mean difference of 3.89 points between oxiracetam and piracetam (95% CI 3.32–4.45), with higher scores in the oxiracetam groups. This estimate is numerically similar to the 4.05-point difference in the present TBI-specific piracetam comparison. The concordance should not be interpreted as independent replication because the two reviews overlap in several primary studies, including Shu et al. (2005), Guo (2009), Sun (2012), Liu (2012), and Duan (2013). These overlapping trials were small, had short follow-up, and generally reported allocation procedures and blinding incompletely. The present review was restricted to acute TBI, separated comparator structures, audited source data and assessment times, excluded unresolved values, and graded certainty by outcome. Because of these design differences and the extensive study overlap, the numerical similarity reflects a largely shared, low-certainty evidence base rather than independent confirmation.

Broader reviews of pharmacological treatment after acquired brain injury also support a cautious interpretation. El Sayed et al. (2018) identified only limited evidence for piracetam among neuroprotective treatments after TBI and concluded that studies with stronger methods were needed. A later meta-analysis of pharmacotherapy for cognitive impairment after acquired brain injury found a small cognitive benefit of methylphenidate in TBI but limited evidence for other agents (van der Veen et al., 2024). Oxiracetam was not evaluated because eligible interventions were restricted to agents registered with the U.S. Food and Drug Administration and/or European Medicines Agency. Accordingly, the oxiracetam trials conducted in China were outside that review’s prespecified evidence base.

LOCATE provides complementary rather than confirmatory evidence. At day 90, the adjusted MMSE differences for L-oxiracetam were 0.71 points relative to placebo and 0.48 points relative to racemic oxiracetam, and both confidence intervals included zero. By contrast, the adjusted increase in the broader Loewenstein Occupational Therapy Cognitive Assessment score was 8.97 points greater with L-oxiracetam than with placebo and 4.54 points greater with L-oxiracetam than with racemic oxiracetam (Liu et al., 2025). This pattern only partly aligns with the higher short-term post-treatment MMSE scores in the earlier trials. LOCATE evaluated a stereoisomeric formulation, enrolled a population in which more than 90% of participants had mild TBI, used a broader cognitive instrument, analyzed change from baseline, and assessed outcomes at 90 days. The earlier trials primarily evaluated short-course racemic oxiracetam and reported unadjusted endpoint scores. These bodies of evidence therefore address related but non-equivalent questions, and the pooled MMSE estimates should not be extrapolated to broad or durable cognitive benefit.

The substantial heterogeneity in the balanced-background MMSE, Barthel Index, and GCS analyses may reflect clinically important differences among studies. Injury severity, formulation, dose, route, treatment duration, time from injury to treatment, background care, and assessment time differed across studies. Most trials reported endpoint values rather than baseline-adjusted changes, so residual baseline imbalance may also have influenced estimates in small samples. Experimental studies suggest that oxiracetam may affect cholinergic and glutamatergic signaling, synaptic plasticity, neuronal metabolism, and neuroinflammatory pathways (Gouliaev and Senning, 1994; Youn et al., 2023). These mechanisms were not tested in the included clinical trials, however, and biological plausibility cannot distinguish a drug effect from natural recovery or the effects of surgery, rehabilitation, and concomitant treatment.

Safety remains particularly uncertain. The earlier trials conducted in China rarely described prospective surveillance, causality assessment, or complete arm-specific event counts. The absence of a statistically significant difference in the pooled safety analysis should therefore not be interpreted as evidence of equivalent safety. In LOCATE, the overall incidence of adverse events was numerically similar across groups and no serious adverse events were considered treatment related, but treatment-related adverse events were most frequent with racemic oxiracetam and differed across the three groups (Liu et al., 2025). Even this substantially larger trial was not designed to exclude rare or delayed harms. Future studies should report all adverse events, serious and treatment-related events, treatment discontinuations, deaths, and prespecified organ-specific outcomes separately.

This review has several strengths. It searched major Chinese and international databases and trial registries, separated comparator groups and outcome domains, evaluated the balance of co-interventions, audited study-level regimens and numerical data against the source reports, excluded unverifiable or internally inconsistent values rather than applying assumption-based corrections, and applied GRADE to the primary analyses. Important limitations nevertheless remain. Most earlier trials were small, single-center studies with inadequate reporting of allocation concealment, blinding, protocol registration, and safety monitoring. Clinical regimens and assessment schedules varied substantially, and most continuous outcomes were reported as post-treatment values rather than baseline-adjusted changes. Some reports did not clearly distinguish SDs from other dispersion measures. Although all five functional-outcome reports used a 0–100 Barthel Index with higher scores indicating better function, the exact versions were not reported. The concentration of the evidence in China, predominance of short-term assessment, limited number of studies within clinically coherent strata, and attrition in LOCATE limit confidence in the effect estimates and their generalizability.

Current guidance does not make an oxiracetam-specific recommendation and instead emphasizes early, structured, or individualized rehabilitation appropriate to the target population (Chinese Medical Association Neurosurgery Branch and Chinese Neurosurgical Critical Care Collaboration Group, 2017; Yang et al., 2022; Bayley et al., 2023; U.S. Department of Veterans Affairs and Department of Defense, 2021). Against this background, the present findings should be regarded as a possible signal of short-term efficacy, not as evidence supporting routine prescribing. Future trials should be prospectively registered and use concealed randomization, blinded outcome assessment where feasible, clearly balanced background care, standardized multidomain cognitive batteries, validated functional and quality-of-life outcomes, baseline-adjusted analyses, and follow-up extending beyond the acute treatment period. Complete safety surveillance is essential. Such trials are needed to determine whether short-term score differences translate into sustained, patient-important benefit.

## 5 Conclusion

Among randomized trials conducted in China, oxiracetam was associated with higher short-term MMSE scores than comparator regimens; some comparisons also showed lower NIHSS scores and higher Barthel Index or GCS scores. However, risk of bias, substantial clinical and statistical heterogeneity, short follow-up, and incomplete safety reporting limit confidence in these estimates. The current evidence neither establishes a durable clinical benefit nor supports routine use of oxiracetam after TBI. Well-designed, prospectively registered randomized trials using standardized cognitive, functional, and safety outcomes and longer follow-up are needed.

## Data Availability

The original contributions presented in the study are included in the article/Supplementary Material, further inquiries can be directed to the corresponding author.

## References

[B1] Bai L. (2015). Effect analysis of oxiracetam for craniocerebral injury. J. Clin. Med. Lit. 2, 5520–5521. 10.16281/j.cnki.jocml.2015.26.120

[B2] Bayley M. T. Janzen S. Harnett A. Teasell R. Patsakos E. Marshall S. (2023). INCOG 2.0 guidelines for cognitive rehabilitation following traumatic brain injury: methods, overview, and principles. J. Head. Trauma Rehabil. 38, 7–23. 10.1097/HTR.0000000000000838 36594856

[B3] Blennow K. Brody D. L. Kochanek P. M. Levin H. McKee A. Ribbers G. M. (2016). Traumatic brain injuries. Nat. Rev. Dis. Prim. 2, 16084. 10.1038/nrdp.2016.84 27853132

[B4] Chen X. H. Wu R. H. Chen G. Y. Feng W. W. Hu Z. H. Hu W. K. (2012). Clinical efficacy and safety of injectable oxiracetam for craniocerebral injury with neurological deficit. China Mod. Med 19, 56–57. 10.3969/j.issn.1674-4721.2012.15.030

[B5] Chen X. Jiang G. Liu X. (2014). Efficacy of oxiracetam for traumatic brain injury. China Health Ind. 11, 113–114. 10.16659/j.cnki.1672-5654.2014.31.063

[B6] Cheng Y. Liu Z. (2020). Effects of oxiracetam in patients with severe craniocerebral injury. Chin. J. Clin. Pharm. 29, 16–20. 10.19577/j.1007-4406.2020.01.004

[B7] Chinese Medical Association Neurosurgery Branch, and Chinese Neurosurgical Critical Care Collaboration Group (2017). Expert consensus on early rehabilitation management of severe traumatic brain injury in China. Natl. Med. J. China 97 (21), 1615–1623. 10.3760/cma.j.issn.0376-2491.2017.21.006

[B8] Cochrane Collaboration (2020). Review Manager 5 (RevMan 5) [computer program], version 5.4.1. Copenhagen: The Cochrane Collaboration.

[B9] DerSimonian R. Laird N. (1986). Meta-analysis in clinical trials. Control. Clin. Trials 7, 177–188. 10.1016/0197-2456(86)90046-2 3802833

[B10] Dewan M. C. Rattani A. Gupta S. Baticulon R. E. Hung Y.-C. Punchak M. (2019). Estimating the global incidence of traumatic brain injury. J. Neurosurg. 130, 1080–1097. 10.3171/2017.10.JNS17352 29701556

[B11] Duan X. (2013). Effects of oxiracetam injection on neurological deficit, memory, and intellectual impairment after moderate traumatic brain injury. Pract. J. Card. Cereb. Pneum. Vasc. Dis. 21, 79–80. 10.3969/j.issn.1008-5971.2013.09.040

[B12] El Sayed I. Zaki A. Fayed A. M. Shehata G. M. Abdelmonem S. (2018). A meta-analysis of the effect of different neuroprotective drugs in management of patients with traumatic brain injury. Neurosurg. Rev. 41, 427–438. 10.1007/s10143-016-0775-y 27539610

[B13] Fan Y. (2016). Efficacy of oxiracetam for neurological deficit caused by craniocerebral injury. Chin. J. Pract. Nerv. Dis. 19, 129–130. 10.3969/j.issn.1673-5110.2016.08.084

[B14] Gouliaev A. H. Senning A. (1994). Piracetam and other structurally related nootropics. Brain Res. Rev. 19, 180–222. 10.1016/0165-0173(94)90011-6 8061686

[B15] Guo X. (2009). Clinical study of oxiracetam injection in moderate-to-severe acute brain injury. China Mod. Med. 16, 42–43. 10.3969/j.issn.1674-4721.2009.19.026

[B16] Guo Y. Sun X. (2015). Effect of oxiracetam on tumor necrosis factor-α in patients with severe traumatic brain injury. J. Hainan Med. Univ. 21, 1222–1224. 10.13210/j.cnki.jhmu.20150313.003

[B17] Guo X. Wang R. (2016). Effect of oxiracetam on serum inflammatory mediators in patients with severe traumatic brain injury. Chin. J. Pract. Nerv. Dis. 19, 45–47. 10.3969/j.issn.1673-5110.2016.05.026

[B18] Guyatt G. H. Oxman A. D. Vist G. E. Kunz R. Falck-Ytter Y. Alonso-Coello P. (2008). GRADE: an emerging consensus on rating quality of evidence and strength of recommendations. BMJ 336, 924–926. 10.1136/bmj.39489.470347 PMC2335261 18436948

[B19] Higgins J. P. T. Altman D. G. Gøtzsche P. C. Jüni P. Moher D. Oxman A. D. (2011). The cochrane Collaboration's tool for assessing risk of bias in randomised trials. BMJ 343, d5928. 10.1136/bmj.d5928 PMC3196245 22008217

[B20] Huang X. (2012). Efficacy of oxiracetam in older patients with severe traumatic brain injury. Chin. J. Gerontol. 32, 2159–2160. 10.3969/j.issn.1005-9202.2012.10.080

[B21] Liang Q. Liu R. (2016). Randomized controlled study of oxiracetam plus nimodipine in severe cerebral contusion and laceration. Med. Forum 20, 3504–3505. 10.19435/j.1672-1721.2016.25.024

[B22] Liu Z. (2012). Clinical effects of oxiracetam in craniocerebral injury. Strait Pharm. J. 24, 97–98.

[B23] Liu M. (2018). Efficacy of oxiracetam injection for acute craniocerebral injury. J. North. Pharm. 15, 124. 10.3969/j.issn.1672-8351.2018.08.105

[B24] Liu H. Zhang J. (2020). Clinical effects of oxiracetam injection in older patients with craniocerebral injury. Beijing Med. J. 42, 1007–1009. 10.15932/j.0253-9713.2020.10.020

[B25] Liu B. Zeng R. Zhong H. (2014). Clinical observation of oxiracetam in 25 patients with craniocerebral injury. Chin. J. Ethnomed. Ethnopharm. 23, 65.

[B26] Liu T. Wang J. Zhao Z. Jiang W. Zhang M. Yu Y. (2025). Efficacy and safety of L-oxiracetam on cognitive function in patients with traumatic brain injury: a multicentre, randomised, double-blind, phase 3 clinical trial. Signal Transduct. Target. Ther. 10, 401. 10.1038/s41392-025-02492-5 41381424 PMC12698704

[B27] Maas A. I. R. Menon D. K. Adelson P. D. Andelic N. Bell M. J. Belli A. (2017). Traumatic brain injury: integrated approaches to improve prevention, clinical care, and research. Lancet Neurol. 16, 987–1048. 10.1016/S1474-4422(17)30371-X 29122524

[B28] Maas A. I. R. Menon D. K. Manley G. T. Abrams M. Åkerlund C. Andelic N. (2022). Traumatic brain injury: progress and challenges in prevention, clinical care, and research. Lancet Neurol. 21, 1004–1060. 10.1016/S1474-4422(22)00309-X PMC10427240 36183712

[B29] Page M. J. McKenzie J. E. Bossuyt P. M. Boutron I. Hoffmann T. C. Mulrow C. D. (2021). The PRISMA 2020 statement: an updated guideline for reporting systematic reviews. BMJ 372, n71. 10.1136/bmj.n71 33782057 PMC8005924

[B30] Qi J. Jiang X. Feng G. Chen P. (2022). Effect of oxiracetam on rescue outcomes in patients with acute severe craniocerebral injury and traumatic shock. Tianjin Pharm. 34, 20–23. 10.3969/j.issn.1006-5687.2022.03.004

[B31] Shu K. Xi G. Niu H. Dong Z. Yu J. Lei T. (2005). Clinical evaluation of oxiracetam in the treatment of craniocerebral trauma. Chin. J. Neuromed. 4, 474–476. 10.3760/cma.j.issn.1671-8925.2005.05.014

[B32] Si D. Muyiti A. Li T. Chen T. (2015). Clinical Observation Of Oxiracetam combined with nimodipine in severe cerebral contusion and laceration. Drug Clin. 30, 184–187. 10.7501/j.issn.1674-5515.2015.02.016

[B33] Sui C. Hu L. Fan C. Ma S. (2019). Clinical effects of oxiracetam injection and its effects on inflammation and oxidative stress in older patients with craniocerebral injury. Chin. J. Mult. Organ Dis. Elder. 18, 896–899. 10.11915/j.issn.1671-5403.2019.12.187

[B34] Sun X. (2012). Efficacy of oxiracetam injection in acute craniocerebral injury. Med. Innov. China 9, 140–141. 10.3969/j.issn.1674-4985.2012.09.088

[B35] Sun G. (2018). Effect of Oxiracetam Combined With nimodipine in severe cerebral contusion and laceration. World Latest Med. Inf. 18 (74), 125–127. 10.19613/j.cnki.1671-3141.2018.74.095

[B36] Tan M. (2020). Effect of oxiracetam combined with hyperbaric oxygen on prognosis after craniocerebral trauma. J. China Prescr. Drug 18, 91–92. 10.3969/j.issn.1671-945X.2020.09.047

[B37] U.S. Department of Veterans Affairs and Department of Defense (2021). VA/DoD Clinical Practice Guideline for the Management and Rehabilitation of Post-Acute Mild Traumatic Brain Injury, Version 3.0. Washington, DC: U.S. Department of Veterans Affairs and Department of Defense.

[B38] van der Veen R. Königs M. Bakker S. van Iperen A. Peerdeman S. Bet P. M. (2024). Pharmacotherapy to improve cognitive functioning after acquired brain injury: a meta-analysis and meta-regression. Clin. Pharmacol. Ther. 115, 971–987. 10.1002/cpt.3186 38294196

[B39] Wang J. Zhang J. Zhou J. (2016). Systematic review on curative effects of oxiracetam and piracetam in treatment of cerebral organic syndrome. Chin. J. Evid.-Based Cardiovasc. Med. 8, 160–163. 10.3969/j.issn.1674-4055.2016.02.09

[B40] Wang X. Wang F. Jin B. (2018). Efficacy of oxiracetam for cognitive impairment after moderate traumatic brain injury. Chin. J. Ration. Drug Use 15, 1–4. 10.3969/j.issn.2096-3327.2018.07.001

[B41] Wu J. (2014). Clinical observation of oxiracetam for craniocerebral injury. Jilin Med. J. 35, 1222.

[B42] Yang J. Chen D. Sun H. Huang S. Tang L. Luo B. (2013). Efficacy of oxiracetam in severe craniocerebral injury. Med. J. Chin. People's Armed Police Forces 24, 344–345. 10.14010/j.cnki.wjyx.2013.04.014

[B43] Yang X. F. Chen L. Pu J. B. Li Y. S. Cai J. Q. Chen L. K. (2022). Guideline of clinical neurorestorative treatment for brain trauma (2022 China version). J. Neurorestoratol. 10, 100005. 10.1016/j.jnrt.2022.100005

[B44] Yin N. Zhang D. (2014). Clinical efficacy of oxiracetam in older patients with severe craniocerebral injury. Guide China Med. 12, 235–236. 10.15912/j.cnki.gocm.2014.13.191

[B45] Youn D. H. Han S. W. Kim J.-T. Choi H. Lee A. Kim N. (2023). Oxiracetam alleviates anti-inflammatory activity and ameliorates cognitive impairment in the early phase of traumatic brain injury. Acta Neurochir. 165, 2201–2210. 10.1007/s00701-023-05674-8 37380907

[B46] Yu Y. Wang Q. Cai Y. Yao Q. Ding L. He X. (2005). Clinical study of oxiracetam for cognitive impairment after moderate-to-severe brain injury. Chin. J. Neurosurg. Dis. Res. 4, 368–369. 10.3969/j.issn.1671-2897.2005.04.026

[B47] Yun F. Chen X. (2021). Effect of oxiracetam combined with nimodipine in severe cerebral contusion and laceration. China Pract. Med. 16, 93–95. 10.14163/j.cnki.11-5547/r.2021.30.036

[B48] Zhai Z. Zhang J. Tang K. Li Z. (2015). Clinical study of oxiracetam in acute craniocerebral injury. Med. J. West China 27, 834–836. 10.3969/j.issn.1672-3511.2015.06.011

[B49] Zhai J. Wang W. Xiao S. (2018). Effect of oxiracetam on neurological function and serum inflammatory factors in patients with severe traumatic brain injury. Yunnan Med. 39, 500–501. 10.20282/j.cnki.1006-4141.2018.06.008

[B50] Zhang L. (2017). Clinical observation of oxiracetam combined with nimodipine in severe cerebral contusion and laceration. Chin. Community Dr. 33, 25–26. 10.3969/j.issn.1007-614X.2017.3.13

[B51] Zhang G. Shang J. Han Z. Ma X. (2008). Clinical evaluation of oxiracetam for neurological deficit after moderate-to-severe traumatic brain injury. Chin. J. Neurosurg. Dis. Res. 7, 172–173. 10.3969/j.issn.1671-2897.2008.02.023

[B52] Zhou G. Wang P. (2013). Oxiracetam as adjunctive therapy in 48 patients with acute craniocerebral injury. China Pharm. 22, 78. 10.3969/j.issn.1006-4931.2013.15.041

