# Supplementary material for: Efficacy and safety of oxiracetam for cognitive and functional outcomes after acute traumatic brain injury in Chinese populations: a systematic review and meta-analysis of randomized controlled trials
Source: Front Pharmacol. 2026 Sep 21;17:1894256. doi: 10.3389/fphar.2026.1894256 (PMC13635107; doi:10.3389/fphar.2026.1894256)
Supplement: Supplementary file 1 [file Table1.docx]

**Supplementary Table S1. Complete search strategies for bibliographic databases and trial registers**

Searches were conducted from database inception to 28 February 2026. Where an interface did not permit day-level limits, searches were limited to 2026 and records dated after 28 February 2026 were screened manually. Outcome-specific and randomized-trial filters were not applied at the search stage.

| **Source** | **Coverage and limits** | **Search strategy** | **Records retrieved** |
| --- | --- | --- | --- |
| PubMed | Coverage/interface: NCBI PubMed; fields: MeSH, Supplementary Concept, substance/name, title/abstract; publication date from inception to 28 February 2026. | (  "Oxiracetam"[Supplementary Concept] OR "Oxiracetam"[nm] OR oxiracetam[tiab] OR "L-oxiracetam"[tiab] OR "L oxiracetam"[tiab] OR "levorotatory oxiracetam"[tiab] OR "S-oxiracetam"[tiab]  )  AND  (  "Brain Injuries, Traumatic"[Mesh] OR "Craniocerebral Trauma"[Mesh] OR "traumatic brain injur*"[tiab] OR TBI[tiab] OR "craniocerebral traum*"[tiab] OR "craniocerebral injur*"[tiab] OR "cranio-cerebral traum*"[tiab] OR "head injur*"[tiab] OR "brain traum*"[tiab] OR "cerebral traum*"[tiab] OR "brain contusion*"[tiab] OR "cerebral contusion*"[tiab] OR "acute brain injur*"[tiab]  )  AND ("1900/01/01"[Date - Publication] : "2026/02/28"[Date - Publication]) | 10 |
| Cochrane CENTRAL | Coverage/interface: Cochrane Library, Advanced Search, Search Manager; CENTRAL/Trials results; no randomized-trial filter applied; records dated up to 28 February 2026 considered. | #1 MeSH descriptor: [Craniocerebral Trauma] explode all trees  #2 MeSH descriptor: [Brain Injuries, Traumatic] explode all trees  #3 (traumatic NEXT brain NEXT injur*):ti,ab,kw OR TBI:ti,ab,kw OR (craniocerebral NEXT traum*):ti,ab,kw OR (craniocerebral NEXT injur*):ti,ab,kw OR (cranio-cerebral NEXT traum*):ti,ab,kw OR (head NEXT injur*):ti,ab,kw OR (brain NEXT traum*):ti,ab,kw OR (cerebral NEXT traum*):ti,ab,kw OR (brain NEXT contusion*):ti,ab,kw OR (cerebral NEXT contusion*):ti,ab,kw OR (acute NEXT brain NEXT injur*):ti,ab,kw  #4 oxiracetam:ti,ab,kw OR "L-oxiracetam":ti,ab,kw OR "L oxiracetam":ti,ab,kw OR "levorotatory oxiracetam":ti,ab,kw OR "S-oxiracetam":ti,ab,kw  #5 (#1 OR #2 OR #3) AND #4 | 8 |
| CNKI | Coverage/interface: China National Knowledge Infrastructure; professional search; subject field (SU); publication date from inception to 28 February 2026. | (SU='奥拉西坦' OR SU='氧拉西坦' OR SU='L-奥拉西坦' OR SU='左奥拉西坦' OR SU='左旋奥拉西坦')  AND  (SU='颅脑损伤' OR SU='创伤性脑损伤' OR SU='脑外伤' OR SU='颅脑外伤' OR SU='头部外伤' OR SU='头颅外伤' OR SU='急性脑损伤' OR SU='脑损伤' OR SU='重型颅脑损伤' OR SU='中型颅脑损伤' OR SU='中重型颅脑损伤' OR SU='脑挫裂伤') | 126 |
| Wanfang Data | Coverage/interface: Advanced search; topic field; journal articles, dissertations, and conference papers; publication year 1900–2026, with records after 28 February 2026 screened manually. | (奥拉西坦 OR 氧拉西坦 OR L-奥拉西坦 OR L 奥拉西坦 OR 左奥拉西坦 OR 左旋奥拉西坦)  AND  (颅脑损伤 OR 创伤性脑损伤 OR 脑外伤 OR 颅脑外伤 OR 头部外伤 OR 头颅外伤 OR 急性脑损伤 OR 脑损伤 OR 重型颅脑损伤 OR 中型颅脑损伤 OR 中重型颅脑损伤 OR 脑挫裂伤 OR 神经功能缺损) | 171 |
| VIP Database | Coverage/interface: Advanced search; subject-term field; Chinese records only; journal articles, dissertations, and conference papers; 1900-01 to 2026-02; synonym and Chinese-English expansion not used. | (奥拉西坦 OR 氧拉西坦 OR L-奥拉西坦 OR 左奥拉西坦 OR 左旋奥拉西坦)  AND  (颅脑损伤 OR 创伤性脑损伤 OR 脑外伤 OR 颅脑外伤 OR 头部外伤 OR 头颅外伤 OR 急性脑损伤 OR 脑损伤 OR 重型颅脑损伤 OR 中型颅脑损伤 OR 中重型颅脑损伤 OR 脑挫裂伤) | 365 |
| SinoMed/CBM | Coverage/interface: Chinese Biomedical Literature Service System; Chinese literature database; quick/common-field search; publication year 1900–2026, with records after 28 February 2026 screened manually. | (奥拉西坦 OR 氧拉西坦 OR L-奥拉西坦 OR 左奥拉西坦 OR 左旋奥拉西坦)  AND  (颅脑损伤 OR 创伤性脑损伤 OR 脑外伤 OR 颅脑外伤 OR 头部外伤 OR 头颅外伤 OR 急性脑损伤 OR 脑损伤 OR 重型颅脑损伤 OR 中型颅脑损伤 OR 中重型颅脑损伤 OR 脑挫裂伤 OR 颅脑创伤) | 37 |
| ClinicalTrials.gov | Coverage/interface: Find Studies search; Condition/disease and Other terms fields; all study statuses; records first posted on or before 28 February 2026 considered. | Condition/disease: traumatic brain injury OR head injury OR brain injury OR craniocerebral trauma  Other terms: oxiracetam OR "L-oxiracetam" OR "L oxiracetam" OR levorotatory oxiracetam | 1 |
| WHO ICTRP | Coverage/interface: WHO International Clinical Trials Registry Platform Search Portal; free-text search; all phases; records registered on or before 28 February 2026 considered. | Free-text search: oxiracetam | 5 |
| ChiCTR | Coverage/interface: Chinese Clinical Trial Registry trial search; registration status unrestricted; records registered on or before 28 February 2026 considered. | 注册题目/检索试验: 奥拉西坦 | 5 |
| Chinese Drug Clinical Trial Registration and Information Disclosure Platform | Coverage/interface: Trial public query platform; registration status unrestricted; records registered on or before 28 February 2026 considered. | 检索词: 奥拉西坦 | 6 |

Abbreviations: CBM, Chinese Biomedical Literature Database; CENTRAL, Cochrane Central Register of Controlled Trials; ChiCTR, Chinese Clinical Trial Registry; CNKI, China National Knowledge Infrastructure; ICTRP, International Clinical Trials Registry Platform; VIP, Chinese Science and Technology Journal Database.

Records retrieved: bibliographic databases, n = 717; trial registers, n = 17.
