# Supplementary material for: Efficacy and safety of oxiracetam for cognitive and functional outcomes after acute traumatic brain injury in Chinese populations: a systematic review and meta-analysis of randomized controlled trials
Source: Front Pharmacol. 2026 Sep 21;17:1894256. doi: 10.3389/fphar.2026.1894256 (PMC13635107; doi:10.3389/fphar.2026.1894256)
Supplement: Supplementary file 2 [file Table2.docx]

**Supplementary Table S2. Full-text reports excluded after eligibility assessment, with reasons**

*Note. This table lists reports excluded after eligibility assessment. Reports not retrieved before full-text assessment are recorded in the PRISMA flow diagram and are not counted in this table.*

| **No.** | **Report title** | **Authors** | **Year** | **Source / registry** | **Exclusion category** | **Reason for exclusion** |
| --- | --- | --- | --- | --- | --- | --- |
| 1 | 醒脑静注射液联合奥拉西坦注射液对脑外伤患者神经功能的影响及预后分析 | 张继禹; 周康 | 2025 | CNKI; VIP; Wanfang | Intervention/co-intervention mismatch or imbalance | The experimental regimen included Xingnaojing injection in addition to oxiracetam, so the effect of oxiracetam could not be isolated. |
| 2 | 奥拉西坦联合左乙拉西坦治疗脑外伤后癫痫的疗效及对血清S100β、NSE水平的影响 | 徐兴梦; 张良明; 刘巧; 顾永仕; 李海龙; 袁晓燕 | 2025 | CNKI; VIP; Wanfang | Intervention/co-intervention mismatch or imbalance | The study focused on post-traumatic epilepsy and included levetiracetam as an unbalanced co-intervention. |
| 3 | 通窍活血汤辅助治疗轻中型颅脑损伤后认知障碍效果观察 | 戴金军; 樊兵剑; 吴来徳; 周翔; 文友东 | 2024 | CNKI; VIP; Wanfang | Intervention/co-intervention mismatch or imbalance | The intervention tested Tongqiao Huoxue decoction added to oxiracetam; the independent effect of oxiracetam was not estimable. |
| 4 | 自拟活血开窍汤联合奥拉西坦治疗轻中型颅脑损伤的临床观察 | 唐骥文 | 2024 | CNKI; Wanfang | Intervention/co-intervention mismatch or imbalance | A Chinese herbal formula was combined with oxiracetam, creating an unbalanced co-intervention. |
| 5 | 通窍化瘀醒神汤联合奥拉西坦对重度颅脑损伤患者血清NSE、microRNA-210的影响分析 | 刘雪楠; 王琛; 李文娟 | 2023 | CNKI; VIP; Wanfang | Intervention/co-intervention mismatch or imbalance | The experimental group received a Chinese herbal formula in addition to oxiracetam, making attribution to oxiracetam alone inappropriate. |
| 6 | 低频重复经颅磁刺激联合奥拉西坦对重度颅脑损伤患者认知功能、脑血流及不良情绪的影响 | 常丙林; 常晓赞; 单春格; 周艳宏 | 2023 | CNKI; VIP; Wanfang | Intervention/co-intervention mismatch or imbalance | Low-frequency repetitive transcranial magnetic stimulation was an additional active intervention. |
| 7 | 果糖二磷酸钠联合奥拉西坦治疗急性颅脑损伤的效果及对神经功能恢复的影响 | 董松宝; 王飞 | 2022 | CNKI; VIP; Wanfang | Intervention/co-intervention mismatch or imbalance | Fructose diphosphate was combined with oxiracetam and was not balanced between groups. |
| 8 | 果糖二磷酸钠联合奥拉西坦治疗急性颅脑损伤伴认知障碍临床观察 | 周明锴; 杜航; 张丹丹; 王睿; 宋豆豆; 潘鹏宇; 杨宵鹏 | 2022 | CNKI; VIP; Wanfang | Intervention/co-intervention mismatch or imbalance | Fructose diphosphate was an unbalanced co-intervention. |
| 9 | 奥拉西坦联合醒脑静治疗脑外伤的效果及对神经元损伤的影响 | 孙彦君 | 2022 | CNKI; VIP; Wanfang | Intervention/co-intervention mismatch or imbalance | Xingnaojing was combined with oxiracetam, so the oxiracetam-specific effect could not be separated. |
| 10 | 低温联合奥拉西坦对重型颅脑损伤患者髓鞘碱性蛋白及胶质纤维酸性蛋白表达的影响 | 王光绿; 邓峰; 潘明远; 陈琨; 王百欣; 莫贻敏; 简俊红 | 2021 | CNKI; VIP; Wanfang | Intervention/co-intervention mismatch or imbalance | Therapeutic hypothermia was an additional active intervention in the experimental arm. |
| 11 | 重复经颅磁刺激(rTMS)联合奥拉西坦治疗轻度脑外伤后认知功能障碍的疗效观察 | 孙国生; 丰育功 | 2020 | CNKI | Intervention/co-intervention mismatch or imbalance | Repetitive transcranial magnetic stimulation was combined with oxiracetam and was not balanced between groups. |
| 12 | 果糖二磷酸钠联合奥拉西坦治疗急性颅脑损伤的临床研究 | 崔娟; 袁波; 闫琛; 刘伟 | 2020 | CNKI; VIP; Wanfang | Intervention/co-intervention mismatch or imbalance | Fructose diphosphate was an unbalanced co-intervention. |
| 13 | 奥拉西坦联合红花黄色素治疗颅脑外伤的疗效 | 谢运涛; 张亚磊; 刘沛涛; 乔景尊 | 2020 | CNKI; VIP; Wanfang | Intervention/co-intervention mismatch or imbalance | Safflower yellow pigment was combined with oxiracetam, preventing attribution to oxiracetam alone. |
| 14 | 高压氧联合奥拉西坦对脑外伤后患者的认知功能及相关细胞因子的影响 | 王永楠; 殷杰; 汤畅通 | 2019 | CNKI; VIP; Wanfang | Intervention/co-intervention mismatch or imbalance | Hyperbaric oxygen therapy was an unbalanced co-intervention. |
| 15 | 红花黄色素联合奥拉西坦对颅脑外伤患者神经损伤修复作用、血管收缩舒张的影响及疗效分析 | 唐怀波; 肖顺武; 张永; 申昊; 续岭 | 2019 | CNKI; VIP; Wanfang | Intervention/co-intervention mismatch or imbalance | Safflower yellow pigment was an additional intervention in the experimental regimen. |
| 16 | 醒脑静联合奥拉西坦治疗老年急性颅脑损伤的临床疗效分析 | 王俊杰 | 2017 | CNKI; VIP; Wanfang | Intervention/co-intervention mismatch or imbalance | Xingnaojing was added to oxiracetam and was not used as a balanced background treatment. |
| 17 | 开窍醒神汤联合奥拉西坦对老年重型颅脑损伤患者脑保护作用及炎症反应影响 | 张丙辰; 王军; 孙波; 袁杰; 周俊 | 2017 | CNKI; VIP; Wanfang | Intervention/co-intervention mismatch or imbalance | Kaiqiao Xingshen decoction was an unbalanced co-intervention. |
| 18 | 奥拉西坦联合鼠神经生长因子治疗重型脑外伤的临床观察及其对血清神经元特异性烯醇化酶的影响 | 朱美; 任新海; 陈新; 王琪鸿; 曾明泉; 张何源; 钟文诗 | 2017 | CNKI; VIP; Wanfang | Intervention/co-intervention mismatch or imbalance | Mouse nerve growth factor was combined with oxiracetam and was not balanced between groups. |
| 19 | 鼠神经生长因子联合奥拉西坦治疗重型急性颅脑损伤的临床观察 | 李龙; 杨金庆; 蔡邵帅 | 2015 | CNKI; VIP; Wanfang | Intervention/co-intervention mismatch or imbalance | Mouse nerve growth factor was an additional active co-intervention. |
| 20 | 奥拉西坦联合丙泊酚治疗老年重型颅脑损伤的临床疗效研究 | 金伟盛 | 2015 | CNKI; VIP; Wanfang | Intervention/co-intervention mismatch or imbalance | Propofol was combined with oxiracetam, yielding an unbalanced intervention contrast. |
| 21 | 依达拉奉联合奥拉西坦治疗颅脑损伤临床疗效观察 | 马汝平; 韩丽; 高海英; 赵晓波 | 2015 | CNKI; Wanfang | Intervention/co-intervention mismatch or imbalance | Edaravone was combined with oxiracetam and was not balanced between groups. |
| 22 | 奥拉西坦联合高压氧治疗颅脑损伤疗效观察 | 马铁柱; 张赛; 陈旭义 | 2014 | CNKI; VIP; Wanfang | Intervention/co-intervention mismatch or imbalance | Hyperbaric oxygen therapy was added to oxiracetam and was not a balanced co-intervention. |
| 23 | 高压氧联合奥拉西坦对重型颅脑外伤患者记忆与智能的影响 | 熊浩峰; 程强; 彭礼波; 霍建宏; 何有虎 | 2013 | CNKI; VIP; Wanfang | Intervention/co-intervention mismatch or imbalance | Hyperbaric oxygen therapy was combined with oxiracetam, so the effect of oxiracetam could not be isolated. |
| 24 | 脑活素连用奥拉西坦治疗56例重症脑外伤后综合征临床疗效分析 | 占双凤 | 2013 | CNKI; VIP; Wanfang | Intervention/co-intervention mismatch or imbalance | Cerebrolysin was combined with oxiracetam; the population also involved post-traumatic syndrome. |
| 25 | 醒脑静注射液联合奥拉西坦治疗重型颅脑创伤疗效观察 | 孙艳; 张赛; 韩广; 蒋显锋; 陈旭义 | Not reported | VIP | Intervention/co-intervention mismatch or imbalance | A Xingnaojing plus oxiracetam regimen was evaluated; the treatment contrast did not isolate oxiracetam. |
| 26 | 奥拉西坦联合胞磷胆碱钠干预轻中度颅脑损伤患者认知功能障碍的临床疗效 | 卢经纬; 梁永生 | 2025 | CNKI; VIP; Wanfang | Non-random, quasi-random, or insufficient allocation | Patients were selected randomly but then grouped by treatment regimen rather than by randomized allocation to intervention. |
| 27 | 奥拉西坦联合改良去大骨瓣减压术对颅脑损伤患者免疫功能的影响 | 耿飞; 陈丽 | 2019 | CNKI; VIP; Wanfang | Non-random, quasi-random, or insufficient allocation | Grouping was based on treatment/surgical method, and the surgical co-intervention was not balanced. |
| 28 | 用奥拉西坦治疗创伤性脑损伤的效果探析 | 江振 | 2016 | CNKI; VIP; Wanfang | Non-random, quasi-random, or insufficient allocation | The report was described as retrospective or did not provide adequate randomized allocation information. |
| 29 | 奥拉西坦治疗轻型、中型脑外伤患者的临床疗效分析 | 张勇; 韩晔 | 2015 | CNKI; VIP; Wanfang | Non-random, quasi-random, or insufficient allocation | Randomization was insufficiently described and prespecified outcome data were not adequately extractable. |
| 30 | 奥拉西坦治疗颅脑损伤60例患者的临床研究 | 罗伦; 王辉; 陈川; 宁昕杰; 叶卓鹏; 王辉 | 2014 | CNKI; VIP; Wanfang | Non-random, quasi-random, or insufficient allocation | Allocation by admission odd/even order was quasi-random; the population also included children. |
| 31 | 奥拉西坦注射液治疗急性颅脑损伤的疗效观察 | 沈蓉蓉 | 2013 | CNKI; VIP; Wanfang | Non-random, quasi-random, or insufficient allocation | The report did not provide sufficient information to support randomized allocation. |
| 32 | 奥拉西坦治疗老年重型颅脑损伤患者的效果研究 | 罗林 | 2013 | CNKI; VIP; Wanfang | Non-random, quasi-random, or insufficient allocation | Grouping was not clearly randomized and appeared to be non-randomized. |
| 33 | 奥拉西坦注射液治疗脑损伤临床疗效观察 | 程明东 | 2013 | Wanfang | Non-random, quasi-random, or insufficient allocation | Random allocation and methodological details were insufficiently reported for inclusion as an RCT. |
| 34 | 奥拉西坦治疗轻型颅脑外伤患者记忆与智能障碍 | 方波; 袁鹏; 蔡云红 | 2005 | CNKI; VIP; Wanfang | Non-random, quasi-random, or insufficient allocation | Allocation according to case number was quasi-random and predictable. |
| 35 | 观察研究奥拉西坦与尼莫地平联合治疗重症脑挫裂伤的疗效 | 司晓明; 王春宝; 刘文静 | 2018 | CNKI; VIP; Wanfang | Non-random, quasi-random, or insufficient allocation | Systematic sampling rather than true random allocation was used. |
| 36 | 奥拉西坦治疗颅脑损伤的临床效果观察 | 辛文燕; 司景哲 | 2019 | Wanfang | Non-random, quasi-random, or insufficient allocation | Allocation by odd/even admission order was quasi-random and predictable. |
| 37 | 通窍活血汤合涤痰汤加味方联合奥拉西坦治疗脑外伤后综合征60例 | 林波; 邓红燕; 刘敏 | 2020 | CNKI; VIP; Wanfang | Population/disease outside PICOS | The study involved post-traumatic syndrome and an additional Chinese medicine intervention, outside the acute TBI PICOS. |
| 38 | 注射用鼠神经生长因子联合奥拉西坦治疗高血压脑出血的临床研究 | 于海永; 张颖; 师仰宏 | 2020 | CNKI; VIP; Wanfang | Population/disease outside PICOS | The target condition was hypertensive intracerebral hemorrhage rather than acute traumatic brain injury. |
| 39 | 回神醒脑汤联合奥拉西坦对女性脑外伤后综合征病人sICAM-1、IL-6水平及动脉血流速度的影响 | 杨勇; 黄云; 韦迪岱; 李玻; 李卿卿 | 2020 | CNKI; VIP; Wanfang | Population/disease outside PICOS | The population was post-traumatic syndrome rather than acute TBI, with an additional herbal intervention. |
| 40 | 丁苯酞联合奥拉西坦对重症颅脑损伤继发脑梗死患者炎症因子及凝血功能的影响 | 赵旭; 曹娟 | 2020 | CNKI; VIP; Wanfang | Population/disease outside PICOS | The study focused on secondary cerebral infarction after severe TBI, outside the prespecified PICOS. |
| 41 | 加味解郁通窍汤联合奥拉西坦对脑外伤后综合征患者临床症状、动脉血流速度及血清IL-6、MCP-1、sICAM-1水平的影响 | 姜雷; 徐丽丽; 陈祥涛; 顾海波; 王少阳 | 2019 | CNKI; VIP; Wanfang | Population/disease outside PICOS | The condition was post-traumatic syndrome and the co-intervention was not balanced. |
| 42 | 苦碟子注射液联合奥拉西坦防治重症颅脑损伤继发脑梗死的疗效及对凝血功能及血清炎性因子的影响 | 徐庆; 张立红 | 2018 | CNKI; VIP; Wanfang | Population/disease outside PICOS | The intervention targeted secondary cerebral infarction and used an additional active agent. |
| 43 | 奥拉西坦联合高压氧治疗脑外伤综合征的疗效分析 | 施丽琴; 杨文进; 方琴; 曹利敏 | 2018 | CNKI; VIP; Wanfang | Population/disease outside PICOS | The population was post-traumatic syndrome rather than acute TBI. |
| 44 | 奥拉西坦联合神经节苷脂治疗小儿脑损伤的临床观察 | 孙楠; 刘倩; 高山; 周建超 | 2018 | CNKI; VIP; Wanfang | Population/disease outside PICOS | The study involved pediatric brain injury and an unbalanced ganglioside co-intervention. |
| 45 | 尤瑞克林治疗血管性认知障碍的临床疗效观察 | 祁秀峰 | 2017 | CNKI; Wanfang | Population/disease outside PICOS | The condition was vascular cognitive impairment, not traumatic brain injury. |
| 46 | 奥拉西坦对中度颅脑损伤后认知障碍的疗效分析 | 王向阳 | 2017 | CNKI; VIP; Wanfang | Duplicate or overlapping report | Thesis report overlapped with the included journal article by the same author/topic; the journal report was retained. |
| 47 | 奥拉西坦治疗颅脑损伤引起神经功能缺失的可行性及疗效分析 | 黄俊红; 叶党华; 孟玉丽; 桂志勇; 谭占国 | 2016 | CNKI; VIP; Wanfang | Duplicate or overlapping report | The report appeared to overlap with another included study on oxiracetam for neurological deficit after craniocerebral injury. |
| 48 | 奥拉西坦治疗急性重型颅脑损伤合并创伤性休克效果观察 | 刘卓; 王倩梅; 雒番阳; 龚晓亮; 王彦军; 尹文 | 2016 | CNKI; VIP; Wanfang | Duplicate or overlapping report | The report appeared to overlap with another included study on acute severe TBI with traumatic shock. |
| 49 | 奥拉西坦改善中型颅脑损伤患者神经功能缺失、记忆与智能障碍的疗效分析 | 于万水 | 2013 | CNKI; VIP | Duplicate or overlapping report | The report overlapped with a retained study on middle-grade craniocerebral injury with neurological and cognitive outcomes. |
| 50 | 注射用奥拉西坦治疗颅脑损伤并神经功能缺失的有效性及安全性研究 | 李建科 | 2012 | CNKI; VIP | Duplicate or overlapping report | The report overlapped with a retained trial on oxiracetam for craniocerebral injury with neurological deficit. |
| 51 | 奥拉西坦治疗38例急性颅脑损疗效观察 | 伍三妹; 占双凤 | 2008 | CNKI; VIP; Wanfang | Duplicate or overlapping report | The report appeared to overlap with another 38-case acute craniocerebral injury report and contained inconsistent outcome data. |
| 52 | 奥拉西坦注射液治疗38例急性颅脑损伤的效果分析 | 张恒金 | 2013 | CNKI; VIP; Wanfang | Duplicate or overlapping report | The report appeared to overlap with a 38-case acute craniocerebral injury dataset and contained inconsistent outcome information. |
| 53 | 奥拉西坦对急性轻型脑伤患者功能的影响 | 但炜; 孙晓川; 刘福英; 唐文渊 | 2006 | CNKI; VIP; Wanfang | Non-clinical, review, or non-prespecified outcome | The report focused on functional/electrophysiological outcomes not aligned with the prespecified clinical outcomes for quantitative synthesis. |
| 54 | P300监测对奥拉西坦治疗轻型脑伤的疗效观察 | 张涛; 谢延风; 孙晓川; 唐文渊 | 2006 | CNKI; VIP; Wanfang | Non-clinical, review, or non-prespecified outcome | The primary outcome was P300 monitoring rather than prespecified clinical cognitive or functional scales. |
| 55 | 定量脑电图对奥拉西坦治疗轻中型脑损伤疗效评价 | 谢延风; 张涛; 孙晓川; 但炜; 唐文渊 | 2005 | CNKI; VIP; Wanfang | Non-clinical, review, or non-prespecified outcome | The report primarily evaluated quantitative EEG rather than prespecified clinical endpoints. |
| 56 | 奥拉西坦治疗轻中型脑伤疗效的神经电生理研究 | 张涛 | 2005 | CNKI; VIP; Wanfang | Non-clinical, review, or non-prespecified outcome | The study focused on neurophysiological measures and did not provide suitable prespecified clinical outcome data. |
| 57 | 奥拉西坦对急性轻型脑损伤患者脑电图的影响 | 但炜; 孙晓川; 刘福英; 唐文渊 | 2005 | CNKI; VIP; Wanfang | Non-clinical, review, or non-prespecified outcome | The main outcome was EEG change rather than a prespecified clinical endpoint. |
| 58 | 奥拉西坦注射液对治疗脑外伤的疗效 | 吴继伟 | 2018 | Wanfang | Outcome/data not extractable | The main outcomes were VAS and GOS; no prespecified continuous outcome suitable for the planned meta-analysis was available. |
| 59 | 奥拉西坦治疗脑外伤的疗效分析 | 周朝晖 | 2009 | CNKI; VIP; Wanfang | Outcome/data not extractable | Outcome data and methodological details were insufficient for extraction into the prespecified analyses. |
| 60 | 奥拉西坦治疗轻中型脑伤的临床研究 | 谢延风; 张涛; 孙晓川; 但炜; 唐文渊 | 2006 | CNKI; VIP; Wanfang | Outcome/data not extractable | Available data were not reported in a form suitable for the prespecified meta-analysis. |
| 61 | 奥拉西坦治疗脑外伤患者的临床疗效分析 | 于亲德 | 2013 | Wanfang | Data/design reliability concerns | Reported sample sizes and outcome tables were inconsistent, and the clinical population description was unclear. |
| 62 | 奥拉西坦治疗80例颅脑损伤所致神经功能缺失 | 王成云; 李明超 | 2017 | CNKI; VIP; Wanfang | Data/design reliability concerns | The title/sample size and reported group data were inconsistent, raising concerns about data reliability. |
| 63 | 奥拉西坦对脑外伤炎症状态和认知功能的影响 | 王黎一; 习望; 张时远; 赵梦莎; 张耕源; 曹旭华 | 2025 | CNKI; VIP; Wanfang | Data/design reliability concerns | The study design was observational/non-strictly randomized and contained clinical plus experimental components, limiting eligibility for the RCT synthesis. |
| 64 | 奥拉西坦对中年创伤性脑损伤患者认知障碍的治疗机理及临床疗效研究 | ChiCTR2200059667 | 2022 | ChiCTR | Registered study without publicly available results | A relevant registered study was identified, but no publicly available results were available for extraction. |
| 65 | 注射用左旋奥拉西坦改善颅脑损伤患者记忆/智能障碍的II期探索性临床试验 | CTR20170517 | 2017 | Chinese Drug Clinical Trial Registration and Information Disclosure Platform | Registered study without publicly available results | A phase II registered trial in craniocerebral injury was identified, but no extractable public results were available. |

Summary of exclusion categories: intervention/co-intervention mismatch or imbalance (n=25); non-random, quasi-random, or insufficient allocation (n=11); population/disease outside PICOS (n=9); duplicate or overlapping report (n=7); non-clinical, review, or non-prespecified outcome (n=5); outcome/data not extractable (n=3); data/design reliability concerns (n=3); registered study without publicly available results (n=2).
