# Supplementary material for: Efficacy and safety of oxiracetam for cognitive and functional outcomes after acute traumatic brain injury in Chinese populations: a systematic review and meta-analysis of randomized controlled trials
Source: Front Pharmacol. 2026 Sep 21;17:1894256. doi: 10.3389/fphar.2026.1894256 (PMC13635107; doi:10.3389/fphar.2026.1894256)
Supplement: Supplementary file 3 [file Table3.docx]

**Supplementary Table S3. Detailed evaluation of co-interventions and balanced background therapy in included Chinese randomized trial reports**

Note. This table evaluates whether the planned comparison allowed the effect of oxiracetam to be interpreted within the intended comparator framework. “Probably balanced” indicates that the report described the background treatment as comparable or did not identify an unequal non-oxiracetam co-intervention, while acknowledging that several early reports did not fully itemize routine care. Outcome-level uncertainties in Bai (2015) and Zhai Z. et al. (2015) were handled by excluding unverifiable values from the relevant quantitative syntheses; such values did not inform GRADE.

| **Synthesis category** | **Reports** | **Background/comparator feature** | **Handling** |
| --- | --- | --- | --- |
| Oxiracetam add-on / balanced background therapy vs control | 18 | Routine or otherwise balanced background TBI care; oxiracetam was the planned additional intervention. | Primary quantitative comparison. |
| Oxiracetam vs piracetam | 10 | Both arms received comparable background care; the active nootropic comparator differed by design. | Active-comparator quantitative comparison. |
| Oxiracetam + nimodipine vs nimodipine | 5 | Nimodipine was used as the shared background therapy. | Separate nimodipine-background comparison. |

| **No.** | **Study/report** | **Comparison used in synthesis** | **Experimental arm** | **Control arm** | **Reported background or co-interventions** | **Co-intervention balance judgement** | **Handling in analysis** |
| --- | --- | --- | --- | --- | --- | --- | --- |
| 1 | Qi 2022 | Oxiracetam add-on / balanced background therapy vs control | Oxiracetam added to routine/conventional TBI care. | Routine/conventional TBI care without oxiracetam. | Routine emergency/rescue management for severe TBI with traumatic shock was provided as background care; the exact components were not fully itemized. No unequal non-oxiracetam co-intervention was identified from the extracted report. | Probably balanced as reported; full background regimen incompletely specified. | Included in the add-on/balanced-background quantitative synthesis. |
| 2 | Cheng 2020 | Oxiracetam add-on / balanced background therapy vs control | Oxiracetam added to routine/conventional TBI care. | Routine/conventional TBI care without oxiracetam. | Routine/conventional TBI management was described as the background treatment; detailed components were variably reported and generally not fully itemized. No unequal surgery, rehabilitation, hyperbaric oxygen, citicoline, edaravone, ganglioside, traditional Chinese medicine or other cognitive-enhancing co-intervention was identified from the extracted report. | Probably balanced as reported; full background regimen incompletely specified. | Included in the add-on/balanced-background quantitative synthesis. |
| 3 | Liu Haiyan 2020 | Oxiracetam add-on / balanced background therapy vs control | Oxiracetam added to routine/conventional TBI care. | Routine/conventional TBI care without oxiracetam. | Conventional management for elderly TBI/severe TBI was used as background care. Components such as neurosurgical/supportive treatment, intracranial-pressure management, anti-infective therapy, fluid/electrolyte correction and nutritional support were not consistently itemized. No co-intervention was reported as unequally applied between groups. | Probably balanced as reported; full background regimen incompletely specified. | Included in the add-on/balanced-background quantitative synthesis. |
| 4 | Tan 2020 | Oxiracetam add-on / balanced background therapy vs control | Oxiracetam added to hyperbaric oxygen and routine care. | Hyperbaric oxygen and routine care without oxiracetam. | Hyperbaric oxygen was the shared background intervention in both groups, together with routine TBI care. No additional unequal neuroprotective, rehabilitation, traditional Chinese medicine, citicoline, edaravone or ganglioside intervention was identified from the extracted report. | Probably balanced for hyperbaric oxygen background. | Included in the add-on/balanced-background quantitative synthesis. |
| 5 | Sui 2019 | Oxiracetam add-on / balanced background therapy vs control | Oxiracetam added to routine/conventional TBI care. | Routine/conventional TBI care without oxiracetam. | Conventional management for elderly TBI/severe TBI was used as background care. Components such as neurosurgical/supportive treatment, intracranial-pressure management, anti-infective therapy, fluid/electrolyte correction and nutritional support were not consistently itemized. No co-intervention was reported as unequally applied between groups. | Probably balanced as reported; full background regimen incompletely specified. | Included in the add-on/balanced-background quantitative synthesis. |
| 6 | Wang Xiangyang 2018 | Oxiracetam add-on / balanced background therapy vs control | Oxiracetam added to routine/conventional TBI care. | Routine/conventional TBI care without oxiracetam. | Routine/conventional TBI management was described as the background treatment; detailed components were variably reported and generally not fully itemized. No unequal surgery, rehabilitation, hyperbaric oxygen, citicoline, edaravone, ganglioside, traditional Chinese medicine or other cognitive-enhancing co-intervention was identified from the extracted report. | Probably balanced as reported; full background regimen incompletely specified. | Included in the add-on/balanced-background quantitative synthesis. |
| 7 | Zhai Jingguang 2018 | Oxiracetam add-on / balanced background therapy vs control | Oxiracetam added to routine/conventional TBI care. | Routine/conventional TBI care without oxiracetam. | Routine/conventional TBI management was described as the background treatment; detailed components were variably reported and generally not fully itemized. No unequal surgery, rehabilitation, hyperbaric oxygen, citicoline, edaravone, ganglioside, traditional Chinese medicine or other cognitive-enhancing co-intervention was identified from the extracted report. | Probably balanced as reported; full background regimen incompletely specified. | Included in the add-on/balanced-background quantitative synthesis. |
| 8 | Fan 2016 | Oxiracetam add-on / balanced background therapy vs control | Oxiracetam added to routine/conventional TBI care. | Routine/conventional TBI care without oxiracetam. | Routine/conventional TBI management was described as the background treatment; detailed components were variably reported and generally not fully itemized. No unequal surgery, rehabilitation, hyperbaric oxygen, citicoline, edaravone, ganglioside, traditional Chinese medicine or other cognitive-enhancing co-intervention was identified from the extracted report. | Probably balanced as reported; full background regimen incompletely specified. | Included in the add-on/balanced-background quantitative synthesis. |
| 9 | Guo Xiaoming 2016 | Oxiracetam add-on / balanced background therapy vs control | Oxiracetam added to routine/conventional TBI care. | Routine/conventional TBI care without oxiracetam. | Routine/conventional TBI management was described as the background treatment; detailed components were variably reported and generally not fully itemized. No unequal surgery, rehabilitation, hyperbaric oxygen, citicoline, edaravone, ganglioside, traditional Chinese medicine or other cognitive-enhancing co-intervention was identified from the extracted report. | Probably balanced as reported; full background regimen incompletely specified. | Included in the add-on/balanced-background quantitative synthesis. |
| 10 | Bai 2015 | Oxiracetam add-on / balanced background therapy vs control | Oxiracetam added to routine/conventional TBI care. | Routine/conventional TBI care without oxiracetam. | Routine/conventional TBI management was described as the background treatment; detailed components were variably reported and generally not fully itemized. No unequal surgery, rehabilitation, hyperbaric oxygen, citicoline, edaravone, ganglioside, traditional Chinese medicine or other cognitive-enhancing co-intervention was identified from the extracted report. | Probably balanced as reported; full background regimen incompletely specified. | Included in comparator-specific syntheses. The unnamed mental-status and neurological-deficit scales were excluded from the strict MMSE and NIHSS primary analyses and included only in sensitivity analyses; these sensitivity analyses did not inform GRADE. |
| 11 | Guo Yongxiang 2015 | Oxiracetam add-on / balanced background therapy vs control | Oxiracetam added to routine/conventional TBI care. | Routine/conventional TBI care without oxiracetam. | Routine/conventional TBI management was described as the background treatment; detailed components were variably reported and generally not fully itemized. No unequal surgery, rehabilitation, hyperbaric oxygen, citicoline, edaravone, ganglioside, traditional Chinese medicine or other cognitive-enhancing co-intervention was identified from the extracted report. | Probably balanced as reported; full background regimen incompletely specified. | Included in the add-on/balanced-background quantitative synthesis. |
| 12 | Zhai Zhongwen 2015 | Oxiracetam add-on / balanced background therapy vs control | Oxiracetam added to routine/conventional TBI care. | Routine/conventional TBI care without oxiracetam. | Routine/conventional TBI management was described as the background treatment; detailed components were variably reported and generally not fully itemized. No unequal surgery, rehabilitation, hyperbaric oxygen, citicoline, edaravone, ganglioside, traditional Chinese medicine or other cognitive-enhancing co-intervention was identified from the extracted report. | Probably balanced as reported; full background regimen incompletely specified. | Included in comparator-specific syntheses for outcomes with verifiable data. GCS was excluded from the strict primary analysis because the printed control-group SD of 11.17 was incompatible with the scale range; no decimal-place correction or imputation was used. The control-arm adverse-event count was not reported, so the safety outcome was not quantitatively estimable. |
| 13 | Wu Jianlun 2014 | Oxiracetam add-on / balanced background therapy vs control | Oxiracetam added to routine/conventional TBI care. | Routine/conventional TBI care without oxiracetam. | Routine/conventional TBI management was described as the background treatment; detailed components were variably reported and generally not fully itemized. No unequal surgery, rehabilitation, hyperbaric oxygen, citicoline, edaravone, ganglioside, traditional Chinese medicine or other cognitive-enhancing co-intervention was identified from the extracted report. | Probably balanced as reported; full background regimen incompletely specified. | Included in the add-on/balanced-background quantitative synthesis. |
| 14 | Yin 2014 | Oxiracetam add-on / balanced background therapy vs control | Oxiracetam added to routine/conventional TBI care. | Routine/conventional TBI care without oxiracetam. | Conventional management for elderly TBI/severe TBI was used as background care. Components such as neurosurgical/supportive treatment, intracranial-pressure management, anti-infective therapy, fluid/electrolyte correction and nutritional support were not consistently itemized. No co-intervention was reported as unequally applied between groups. | Probably balanced as reported; full background regimen incompletely specified. | Included in the add-on/balanced-background quantitative synthesis. |
| 15 | Zhou 2013 | Oxiracetam add-on / balanced background therapy vs control | Oxiracetam added to routine/conventional TBI care. | Routine/conventional TBI care without oxiracetam. | Routine/conventional TBI management was described as the background treatment; detailed components were variably reported and generally not fully itemized. No unequal surgery, rehabilitation, hyperbaric oxygen, citicoline, edaravone, ganglioside, traditional Chinese medicine or other cognitive-enhancing co-intervention was identified from the extracted report. | Probably balanced as reported; full background regimen incompletely specified. | Included in the add-on/balanced-background quantitative synthesis. |
| 16 | Chen Xuehua 2012 | Oxiracetam add-on / balanced background therapy vs control | Oxiracetam added to routine/conventional TBI care. | Routine/conventional TBI care without oxiracetam. | Routine/conventional TBI management was described as the background treatment; detailed components were variably reported and generally not fully itemized. No unequal surgery, rehabilitation, hyperbaric oxygen, citicoline, edaravone, ganglioside, traditional Chinese medicine or other cognitive-enhancing co-intervention was identified from the extracted report. | Probably balanced as reported; full background regimen incompletely specified. | Included in the add-on/balanced-background quantitative synthesis. |
| 17 | Huang 2012 | Oxiracetam add-on / balanced background therapy vs control | Oxiracetam added to routine/conventional TBI care. | Routine/conventional TBI care without oxiracetam. | Conventional management for elderly TBI/severe TBI was used as background care. Components such as neurosurgical/supportive treatment, intracranial-pressure management, anti-infective therapy, fluid/electrolyte correction and nutritional support were not consistently itemized. No co-intervention was reported as unequally applied between groups. | Probably balanced as reported; full background regimen incompletely specified. | Included in the add-on/balanced-background quantitative synthesis. |
| 18 | Yu Yigang 2005 | Oxiracetam add-on / balanced background therapy vs control | Oxiracetam added to routine/conventional TBI care. | Routine/conventional TBI care without oxiracetam. | Conventional management included rehabilitation/physical therapy, hyperbaric oxygen and language training as reported in the trial context; oxiracetam was the planned additional intervention. Other neuroprotective or cognitive-enhancing co-interventions were not reported as unequal between groups. | Probably balanced as reported; full background regimen incompletely specified. | Included in the add-on/balanced-background quantitative synthesis. |
| 19 | Liu Min 2018 | Oxiracetam vs piracetam (active-comparator comparison) | Oxiracetam plus the same usual/background treatment as the control arm. | Piracetam plus the same usual/background treatment as the experimental arm. | Usual/basic TBI care was described as comparable between arms; the planned pharmacological difference was oxiracetam versus piracetam. Additional surgery, rehabilitation, hyperbaric oxygen, citicoline, edaravone, ganglioside, traditional Chinese medicine or other cognitive-enhancing treatment was not reported as imbalanced. | Probably balanced for usual/background care; active comparator differed by design. | Included only in the active-comparator meta-analysis; not pooled with usual-care add-on trials. |
| 20 | Chen Xu 2014 | Oxiracetam vs piracetam (active-comparator comparison) | Oxiracetam plus the same usual/background treatment as the control arm. | Piracetam plus the same usual/background treatment as the experimental arm. | Usual/basic TBI care was described as comparable between arms; the planned pharmacological difference was oxiracetam versus piracetam. Additional surgery, rehabilitation, hyperbaric oxygen, citicoline, edaravone, ganglioside, traditional Chinese medicine or other cognitive-enhancing treatment was not reported as imbalanced. | Probably balanced for usual/background care; active comparator differed by design. | Included only in the active-comparator meta-analysis; not pooled with usual-care add-on trials. |
| 21 | Liu Bin 2014 | Oxiracetam vs piracetam (active-comparator comparison) | Oxiracetam plus the same usual/background treatment as the control arm. | Piracetam plus the same usual/background treatment as the experimental arm. | Usual/basic TBI care was described as comparable between arms; the planned pharmacological difference was oxiracetam versus piracetam. Additional surgery, rehabilitation, hyperbaric oxygen, citicoline, edaravone, ganglioside, traditional Chinese medicine or other cognitive-enhancing treatment was not reported as imbalanced. | Probably balanced for usual/background care; active comparator differed by design. | Included only in the active-comparator meta-analysis; not pooled with usual-care add-on trials. |
| 22 | Duan 2013 | Oxiracetam vs piracetam (active-comparator comparison) | Oxiracetam plus the same usual/background treatment as the control arm. | Piracetam plus the same usual/background treatment as the experimental arm. | Usual/basic TBI care was described as comparable between arms; the planned pharmacological difference was oxiracetam versus piracetam. Additional surgery, rehabilitation, hyperbaric oxygen, citicoline, edaravone, ganglioside, traditional Chinese medicine or other cognitive-enhancing treatment was not reported as imbalanced. | Probably balanced for usual/background care; active comparator differed by design. | Included only in the active-comparator meta-analysis; not pooled with usual-care add-on trials. |
| 23 | Yang 2013 | Oxiracetam vs piracetam (active-comparator comparison) | Oxiracetam plus the same usual/background treatment as the control arm. | Piracetam plus the same usual/background treatment as the experimental arm. | Usual/basic TBI care was described as comparable between arms; the planned pharmacological difference was oxiracetam versus piracetam. Additional surgery, rehabilitation, hyperbaric oxygen, citicoline, edaravone, ganglioside, traditional Chinese medicine or other cognitive-enhancing treatment was not reported as imbalanced. | Probably balanced for usual/background care; active comparator differed by design. | Included only in the active-comparator meta-analysis; not pooled with usual-care add-on trials. |
| 24 | Liu Zhongyuan 2012 | Oxiracetam vs piracetam (active-comparator comparison) | Oxiracetam plus the same usual/background treatment as the control arm. | Piracetam plus the same usual/background treatment as the experimental arm. | Usual/basic TBI care was described as comparable between arms; the planned pharmacological difference was oxiracetam versus piracetam. Additional surgery, rehabilitation, hyperbaric oxygen, citicoline, edaravone, ganglioside, traditional Chinese medicine or other cognitive-enhancing treatment was not reported as imbalanced. | Probably balanced for usual/background care; active comparator differed by design. | Included only in the active-comparator meta-analysis; not pooled with usual-care add-on trials. |
| 25 | Sun Xiuhong 2012 | Oxiracetam vs piracetam (active-comparator comparison) | Oxiracetam plus the same usual/background treatment as the control arm. | Piracetam plus the same usual/background treatment as the experimental arm. | Usual/basic TBI care was described as comparable between arms; the planned pharmacological difference was oxiracetam versus piracetam. Additional surgery, rehabilitation, hyperbaric oxygen, citicoline, edaravone, ganglioside, traditional Chinese medicine or other cognitive-enhancing treatment was not reported as imbalanced. | Probably balanced for usual/background care; active comparator differed by design. | Included only in the active-comparator meta-analysis; not pooled with usual-care add-on trials. |
| 26 | Guo Xiaofeng 2009 | Oxiracetam vs piracetam (active-comparator comparison) | Oxiracetam plus the same usual/background treatment as the control arm. | Piracetam plus the same usual/background treatment as the experimental arm. | Usual/basic TBI care was described as comparable between arms; the planned pharmacological difference was oxiracetam versus piracetam. Additional surgery, rehabilitation, hyperbaric oxygen, citicoline, edaravone, ganglioside, traditional Chinese medicine or other cognitive-enhancing treatment was not reported as imbalanced. | Probably balanced for usual/background care; active comparator differed by design. | Included only in the active-comparator meta-analysis; not pooled with usual-care add-on trials. |
| 27 | Zhang Guoqing 2008 | Oxiracetam vs piracetam (active-comparator comparison) | Oxiracetam plus the same usual/background treatment as the control arm. | Piracetam plus the same usual/background treatment as the experimental arm. | Usual/basic TBI care was described as comparable between arms; the planned pharmacological difference was oxiracetam versus piracetam. Additional surgery, rehabilitation, hyperbaric oxygen, citicoline, edaravone, ganglioside, traditional Chinese medicine or other cognitive-enhancing treatment was not reported as imbalanced. | Probably balanced for usual/background care; active comparator differed by design. | Included only in the active-comparator meta-analysis; not pooled with usual-care add-on trials. |
| 28 | Shu Kai 2005 | Oxiracetam vs piracetam (active-comparator comparison) | Oxiracetam plus the same usual/background treatment as the control arm. | Piracetam plus the same usual/background treatment as the experimental arm. | Usual/basic TBI care was described as comparable between arms; the planned pharmacological difference was oxiracetam versus piracetam. Additional surgery, rehabilitation, hyperbaric oxygen, citicoline, edaravone, ganglioside, traditional Chinese medicine or other cognitive-enhancing treatment was not reported as imbalanced. | Probably balanced for usual/background care; active comparator differed by design. | Included only in the active-comparator meta-analysis; not pooled with usual-care add-on trials. |
| 29 | Yun Feifei 2021 | Oxiracetam + nimodipine vs nimodipine (balanced nimodipine-background comparison) | Oxiracetam plus nimodipine and routine care. | Nimodipine and routine care without oxiracetam. | Nimodipine was administered as the common background treatment in both groups, in addition to routine TBI care. The planned difference was the addition of oxiracetam. Other background care was incompletely specified but not reported as unequal. | Probably balanced for nimodipine background; other routine care not fully specified. | Included only in the separate nimodipine-background comparison; not pooled with other comparator types. |
| 30 | Sun Gang 2018 | Oxiracetam + nimodipine vs nimodipine (balanced nimodipine-background comparison) | Oxiracetam plus nimodipine and routine care. | Nimodipine and routine care without oxiracetam. | Nimodipine was administered as the common background treatment in both groups, in addition to routine TBI care. The planned difference was the addition of oxiracetam. Other background care was incompletely specified but not reported as unequal. | Probably balanced for nimodipine background; other routine care not fully specified. | Included only in the separate nimodipine-background comparison; not pooled with other comparator types. |
| 31 | Zhang Lin 2017 | Oxiracetam + nimodipine vs nimodipine (balanced nimodipine-background comparison) | Oxiracetam plus nimodipine and routine care. | Nimodipine and routine care without oxiracetam. | Nimodipine was administered as the common background treatment in both groups, in addition to routine TBI care. The planned difference was the addition of oxiracetam. Other background care was incompletely specified but not reported as unequal. | Probably balanced for nimodipine background; other routine care not fully specified. | Included only in the separate nimodipine-background comparison; not pooled with other comparator types. |
| 32 | Liang 2016 | Oxiracetam + nimodipine vs nimodipine (balanced nimodipine-background comparison) | Oxiracetam plus nimodipine and routine care. | Nimodipine and routine care without oxiracetam. | Nimodipine was administered as the common background treatment in both groups, in addition to routine TBI care. The planned difference was the addition of oxiracetam. Other background care was incompletely specified but not reported as unequal. | Probably balanced for nimodipine background; other routine care not fully specified. | Included only in the separate nimodipine-background comparison; not pooled with other comparator types. |
| 33 | Si Daolin 2015 | Oxiracetam + nimodipine vs nimodipine (balanced nimodipine-background comparison) | Oxiracetam plus nimodipine and routine care. | Nimodipine and routine care without oxiracetam. | Nimodipine was administered as the common background treatment in both groups, in addition to routine TBI care. The planned difference was the addition of oxiracetam. Other background care was incompletely specified but not reported as unequal. | Probably balanced for nimodipine background; other routine care not fully specified. | Included only in the separate nimodipine-background comparison; not pooled with other comparator types. |

Abbreviations: ADL, activities of daily living; CBM, Chinese Biomedical Literature Database; HBO, hyperbaric oxygen; LOCATE, randomized phase 3 L-oxiracetam trial; NIHSS, National Institutes of Health Stroke Scale; RCT, randomized controlled trial; TBI, traumatic brain injury. NR, not reported or not sufficiently itemized.
