# Supplementary material for: Efficacy and safety of oxiracetam for cognitive and functional outcomes after acute traumatic brain injury in Chinese populations: a systematic review and meta-analysis of randomized controlled trials
Source: Front Pharmacol. 2026 Sep 21;17:1894256. doi: 10.3389/fphar.2026.1894256 (PMC13635107; doi:10.3389/fphar.2026.1894256)
Supplement: Supplementary file 4 [file Table4.docx]

**Supplementary Table S4. Summary of findings by comparison and outcome**

Certainty of evidence was assessed using GRADE and is presented using text categories (high, moderate, low, very low) without symbol notation. Chinese trial estimates and ratings refer to strict primary analyses. Continuous outcomes are expressed as mean differences (MDs), and dichotomous outcomes as risk ratios (RRs). Lower NIHSS scores indicate less neurological deficit; higher MMSE, Barthel Index, and GCS scores indicate better scores in their respective domains. GCS was treated as a level-of-consciousness outcome, not a cognitive measure.

A. Strict primary quantitative synthesis of Chinese randomized trial reports

| **Comparison/source** | **Outcome/domain** | **Studies, participants** | **Effect estimate (95% CI)** | **Heterogeneity** | **Certainty (GRADE)** | **Importance** | **Main reasons for rating / comments** |
| --- | --- | --- | --- | --- | --- | --- | --- |
| Oxiracetam add-on or balanced background therapy vs control | MMSE (cognitive function)—strict primary | 10 studies, 798 participants | MD 4.67 higher (3.07 to 6.28 higher) | I² = 92% | Very low | Critical | Risk of bias, very serious inconsistency, indirectness, and possible small-study effects. |
| Oxiracetam add-on or balanced background therapy vs control | Barthel Index (BI) (activities of daily living) | 4 studies, 378 participants | MD 12.79 higher (7.09 to 18.49 higher) | I² = 81% | Very low | Critical | Risk of bias, very serious inconsistency, and indirectness. |
| Oxiracetam add-on or balanced background therapy vs control | NIHSS (neurological deficit)—strict primary | 8 studies, 685 participants | MD 3.22 lower (4.14 to 2.30 lower) | I² = 58% | Low | Critical | Risk of bias and inconsistency; lower scores indicate less neurological deficit. |
| Oxiracetam add-on or balanced background therapy vs control | GCS (level of consciousness)—strict primary | 9 studies, 783 participants | MD 2.64 higher (1.94 to 3.34 higher) | I² = 96% | Very low | Critical | Risk of bias, very serious inconsistency, and indirectness; GCS was not interpreted as cognition. |
| Oxiracetam add-on or balanced background therapy vs control | Investigator-defined response | 5 studies, 558 participants | RR 1.26 (1.16 to 1.37); 251/279 vs 195/279 | I² = 0% | Very low | Important | Risk of bias and indirectness because response definitions were not standardized. |
| Oxiracetam add-on or balanced background therapy vs control | Reported adverse events/adverse reactions—strict primary | 5 studies, 504 participants | RR 1.14 (0.64 to 2.04); 22/253 vs 19/251 | I² = 0% | Very low | Critical | Incomplete and inconsistent harm ascertainment, sparse events, and serious imprecision. |
| Oxiracetam vs piracetam | MMSE (cognitive function) | 8 studies, 739 participants | MD 4.05 higher (3.51 to 4.60 higher) | I² = 0% | Low | Critical | Risk of bias and study limitations despite low statistical heterogeneity. |
| Oxiracetam vs piracetam | Barthel Index (BI) (activities of daily living) | 1 study, 50 participants | MD 21.18 higher (15.02 to 27.34 higher) | Not applicable | Very low | Critical | Single small trial with methodological limitations and imprecision. |
| Oxiracetam vs piracetam | NIHSS (neurological deficit) | 9 studies, 759 participants | MD 3.71 lower (4.54 to 2.87 lower) | I² = 64% | Low | Critical | Risk of bias and inconsistency; lower scores indicate less neurological deficit. |
| Oxiracetam vs piracetam | GCS (level of consciousness) | 8 studies, 775 participants | MD 2.08 higher (1.22 to 2.94 higher) | I² = 90% | Very low | Critical | Risk of bias and very serious inconsistency; GCS was not interpreted as cognition. |
| Oxiracetam vs piracetam | Investigator-defined response | 1 study, 120 participants | RR 1.16 (0.96 to 1.41); 50/60 vs 43/60 | Not applicable | Very low | Important | Single trial, non-standardized composite outcome, and imprecision. |
| Oxiracetam vs piracetam | Reported adverse reactions | 1 study, 95 participants | RR 0.16 (0.02 to 1.30); 1/48 vs 6/47 | Not applicable | Very low | Critical | Single small trial, very few events, and a wide confidence interval. |
| Oxiracetam + nimodipine vs nimodipine | NIHSS (neurological deficit) | 4 studies, 365 participants | MD 4.01 lower (4.52 to 3.49 lower) | I² = 0% | Low | Critical | Risk of bias and indirectness of the shared nimodipine background. |
| Oxiracetam + nimodipine vs nimodipine | GCS (level of consciousness) | 2 studies, 230 participants | MD 4.64 higher (2.27 lower to 11.55 higher) | I² = 100% | Very low | Critical | Risk of bias, extreme inconsistency, and serious imprecision. |
| Oxiracetam + nimodipine vs nimodipine | Investigator-defined response | 4 studies, 405 participants | RR 1.29 (1.17 to 1.42); 179/200 vs 141/205 | I² = 0% | Very low | Important | Risk of bias and indirectness because response definitions were not standardized; one report with internally inconsistent control responder counts was excluded. |

B. Narrative efficacy and structured safety evidence from the LOCATE phase 3 trial

| **Comparison/source** | **Outcome/domain** | **Studies, participants** | **Effect estimate (95% CI)** | **Heterogeneity** | **Certainty (GRADE)** | **Importance** | **Main reasons for rating / comments** |
| --- | --- | --- | --- | --- | --- | --- | --- |
| LOCATE: L-oxiracetam vs placebo | Day-90 LOTCA change | 1 study, 354 participants (235 vs 119) | Adjusted MD 8.97 (5.69 to 12.26) | Not applicable | Moderate | Critical | Downgraded mainly for attrition and missing-data considerations. |
| LOCATE: L-oxiracetam, racemic oxiracetam, and placebo | Structured safety outcomes | 1 study, 590 participants (235/236/119) | Any AE: 155/235, 157/236, 80/119; serious AE: 13/235, 18/236, 15/119; permanent discontinuation due to AE: 1/235, 9/236, 7/119; treatment-related AE: 22/235, 41/236, 11/119 | Not applicable | Moderate | Critical | Single multicenter blinded trial; limited precision for rare events. |

Abbreviations: BI, Barthel Index; CI, confidence interval; GCS, Glasgow Coma Scale; GRADE, Grading of Recommendations Assessment, Development and Evaluation; LOTCA, Loewenstein Occupational Therapy Cognitive Assessment; MD, mean difference; MMSE, Mini-Mental State Examination; MoCA, Montreal Cognitive Assessment; NIHSS, National Institutes of Health Stroke Scale; RCT, randomized controlled trial; RR, risk ratio; TBI, traumatic brain injury.

Note. The LOCATE trial evaluated L-oxiracetam and is summarized separately from pooled estimates of earlier Chinese randomized reports because of differences in intervention form, comparator structure, primary cognitive endpoint and follow-up. Safety findings from LOCATE were included to provide structured contemporary safety evidence.
