# Supplementary material for: Efficacy and safety of oxiracetam for cognitive and functional outcomes after acute traumatic brain injury in Chinese populations: a systematic review and meta-analysis of randomized controlled trials
Source: Front Pharmacol. 2026 Sep 21;17:1894256. doi: 10.3389/fphar.2026.1894256 (PMC13635107; doi:10.3389/fphar.2026.1894256)
Supplement: Supplementary file 5 [file Table5.docx]

**Supplementary Table S5**

**Study-level oxiracetam regimens and outcome assessment timing**

NR, not reported; IV, intravenous. Characteristics were transcribed from the full-text source reports. LOCATE was synthesized separately from the 33 earlier randomized trial reports.

| **ID** | **Study** | **Year** | **Comparison** | **Formulation** | **Dose** | **Frequency** | **Route** | **Injury-to-treatment/start** | **Treatment duration** | **Exact outcome assessment** | **Clinical stratum** | **Source verification** |
| --- | --- | --- | --- | --- | --- | --- | --- | --- | --- | --- | --- | --- |
| 01 | Qi 2022 | 2022 | Oxiracetam add-on / balanced background vs control | Racemic oxiracetam injection | 4 g/day | Once daily | IV infusion | Within 6 h of injury/admission | 14 days | Day 14 | Short-course IV (≤21 days) | Full-text source report |
| 04 | Cheng 2020 | 2020 | Oxiracetam add-on / balanced background vs control | Racemic oxiracetam injection | 4 g/day | Once daily | IV infusion | NR | 12 weeks | End of treatment (12 weeks) | Long-course IV (>21 days) | Full-text source report |
| 02 | Liu Haiyan 2020 | 2020 | Oxiracetam add-on / balanced background vs control | Racemic oxiracetam injection | 4 g/day | Once daily | IV infusion | Within 24 h of admission | 14 days | Admission; days 3, 7, and 14 | Short-course IV (≤21 days) | Full-text source report |
| 03 | Tan 2020 | 2020 | Oxiracetam add-on / balanced background vs control | Racemic oxiracetam capsule | 0.8 g twice daily (1.6 g/day) | Twice daily | Oral | After stabilization; exact injury-to-start time NR | Three 60-day courses (180 days) | End of 180-day treatment | Oral regimen | Full-text source report |
| 05 | Sui 2019 | 2019 | Oxiracetam add-on / balanced background vs control | Racemic oxiracetam injection | 6 g/day; severe cases could receive twice daily | Once daily; severe cases twice daily | IV infusion | NR | 12 days | End of treatment; mortality follow-up | Short-course IV (≤21 days) | Full-text source report |
| 08 | Wang Xiangyang 2018 | 2018 | Oxiracetam add-on / balanced background vs control | Racemic oxiracetam injection | 4 g/day | Once daily | IV infusion | After admission; baseline recorded on admission day 2 | 14 days | Days 7 and 14; day 14 used | Short-course IV (≤21 days) | Full-text source report |
| 06 | Zhai Jingguang 2018 | 2018 | Oxiracetam add-on / balanced background vs control | Racemic oxiracetam injection | 4 g/day | Once daily | IV infusion | NR | 14 days | End of treatment (day 14) | Short-course IV (≤21 days) | Full-text source report |
| 09 | Fan 2016 | 2016 | Oxiracetam add-on / balanced background vs control | Racemic oxiracetam injection | 4 g/day | Once daily | IV infusion | NR | 21 days | End of treatment (3 weeks) | Short-course IV (≤21 days) | Full-text source report |
| 10 | Guo Xiaoming 2016 | 2016 | Oxiracetam add-on / balanced background vs control | Racemic oxiracetam capsule | 0.8 g twice daily (1.6 g/day) | Twice daily | Oral | After admission; exact injury-to-start time NR | 14 days | End of treatment (2 weeks) | Oral regimen | Full-text source report |
| 11 | Bai 2015 | 2015 | Oxiracetam add-on / balanced background vs control | Racemic oxiracetam injection | 0.4 g/day as printed | NR | IV infusion | NR | 20 days | 15-day follow-up after treatment | Short-course IV (≤21 days) | Full-text source report |
| 13 | Guo Yongxiang 2015 | 2015 | Oxiracetam add-on / balanced background vs control | Racemic oxiracetam injection | 4 g/day | Once daily | IV infusion | Postoperative day 1 for patients requiring immediate surgery | 14 days | End of treatment (2 weeks) | Short-course IV (≤21 days) | Full-text source report |
| 12 | Zhai Zhongwen 2015 | 2015 | Oxiracetam add-on / balanced background vs control | Racemic oxiracetam injection | 4 g/day | Once daily | IV infusion | NR | 14 days | GCS at days 7, 14, and 21; excluded from the primary GCS synthesis because the day-21 control dispersion was incompatible with the scale range | Short-course IV (≤21 days) | Full-text source report |
| 16 | Wu Jianlun 2014 | 2014 | Oxiracetam add-on / balanced background vs control | Racemic oxiracetam injection | 4 g/day | Once daily | IV infusion | NR | 21 days | End of treatment (3 weeks) | Short-course IV (≤21 days) | Full-text source report |
| 33 | Yin 2014 | 2014 | Oxiracetam add-on / balanced background vs control | Racemic oxiracetam injection | 4 g/day | Once daily | IV infusion | After admission/standard treatment; exact time NR | 2–3 weeks, individualized | After individualized treatment (2–3 weeks) | Short-course IV (≤21 days) | Full-text source report |
| 18 | Zhou 2013 | 2013 | Oxiracetam add-on / balanced background vs control | Racemic oxiracetam injection | 4 g/day | Once daily | IV infusion | Early use after admission; exact time NR | 21 days | End of treatment (21 days) | Short-course IV (≤21 days) | Full-text source report |
| 20 | Chen Xuehua 2012 | 2012 | Oxiracetam add-on / balanced background vs control | Racemic oxiracetam injection | 4 g/day | Once daily | IV infusion | Mean injury-to-admission time about 5 h | 3 weeks | End of treatment (3 weeks) | Short-course IV (≤21 days) | Full-text source report |
| 21 | Huang 2012 | 2012 | Oxiracetam add-on / balanced background vs control | Racemic oxiracetam injection | 4 g/day; severe cases twice daily | Once daily; severe cases twice daily | IV infusion | NR | 14 days for neurological deficit; 21 days for cognitive impairment | Weeks 3 and 6; week-6 values were used in the quantitative synthesis. | Short-course IV (≤21 days) | Full-text source report |
| 26 | Yu Yigang 2005 | 2005 | Oxiracetam add-on / balanced background vs control | Racemic oxiracetam capsule | 0.8 g three times daily (2.4 g/day) | Three times daily | Oral | After awakening and when oral administration was feasible | 2 months as stated | 1 month; conflicts with stated 2-month course | Oral regimen | Full-text source report |
| 07 | Liu Min 2018 | 2018 | Oxiracetam vs piracetam | Racemic oxiracetam injection | 4 g/day | Once daily | IV infusion | NR | NR | After treatment; exact time NR | IV; duration/assessment time not reported | Full-text source report |
| 14 | Chen Xu 2014 | 2014 | Oxiracetam vs piracetam | Racemic oxiracetam injection | 4 g/day | Once daily | IV infusion | NR | 3 weeks | End of treatment (3 weeks) | Short-course IV (≤21 days) | Full-text source report |
| 15 | Liu Bin 2014 | 2014 | Oxiracetam vs piracetam | Racemic oxiracetam injection | 4 g/day | Once daily | IV infusion | NR | 21 days | End of treatment (21 days) | Short-course IV (≤21 days) | Full-text source report |
| 17 | Duan 2013 | 2013 | Oxiracetam vs piracetam | Racemic oxiracetam injection | 4–8 g/day | Once daily | IV infusion | Immediately after admission/work-up | Three 1-week courses (3 weeks) | End of 3 courses (3 weeks) | Short-course IV (≤21 days) | Full-text source report |
| 19 | Yang 2013 | 2013 | Oxiracetam vs piracetam | Racemic oxiracetam injection | 4 g/day | Once daily | IV infusion | Within 24 h of injury | 21 days | End of treatment (21 days) | Short-course IV (≤21 days) | Full-text source report |
| 23 | Liu Zhongyuan 2012 | 2012 | Oxiracetam vs piracetam | Racemic oxiracetam injection | 4 g/day | Once daily | IV infusion | NR | 2 weeks | 3–6-month follow-up (mean 4.46 months) | Short-course IV with long-term assessment | Full-text source report |
| 22 | Sun Xiuhong 2012 | 2012 | Oxiracetam vs piracetam | Racemic oxiracetam injection | 4 g/day | Once daily | IV infusion | NR | Assessment-specific; 14 days for GCS and 3 weeks for MMSE/NIHSS | GCS day 14; MMSE/NIHSS week 3 | Short-course IV (≤21 days) | Full-text source report |
| 24 | Guo Xiaofeng 2009 | 2009 | Oxiracetam vs piracetam | Racemic oxiracetam injection | 4 g/day | Once daily | IV infusion | NR | 2 weeks for neurological deficit; 3 weeks for cognition | Day 21 for NIHSS, MMSE, and GCS | Short-course IV (≤21 days) | Full-text source report |
| 25 | Zhang Guoqing 2008 | 2008 | Oxiracetam vs piracetam | Racemic oxiracetam injection | 4 g/day | Once daily | IV infusion | NR | 14 days | Day 21 (one week after treatment ended) | Short-course IV (≤21 days) | Full-text source report |
| 27 | Shu Kai 2005 | 2005 | Oxiracetam vs piracetam | Racemic oxiracetam capsule | 0.8 g three times daily (2.4 g/day) | Three times daily | Oral | Injury no more than 72 h before enrollment | 30 days | End of treatment (30 days) | Oral regimen | Full-text source report |
| 32 | Yun Feifei 2021 | 2021 | Oxiracetam + nimodipine vs nimodipine | Racemic oxiracetam injection | 4–6 g/day | Once daily | IV infusion | Postoperative/acute care; exact injury-to-start time NR | 2 weeks | End of treatment (2 weeks) | Short-course IV (≤21 days) | Full-text source report |
| 31 | Sun Gang 2018 | 2018 | Oxiracetam + nimodipine vs nimodipine | Racemic oxiracetam injection | 4 g/day | Once daily | IV infusion | Postoperative/acute care; exact injury-to-start time NR | 14 days | End of treatment (14 days) | Short-course IV (≤21 days) | Full-text source report |
| 30 | Zhang Lin 2017 | 2017 | Oxiracetam + nimodipine vs nimodipine | Racemic oxiracetam injection | 4 g/day | Once daily | IV infusion | Postoperative/acute care; exact injury-to-start time NR | 2 weeks | End of treatment (2 weeks) | Short-course IV (≤21 days) | Full-text source report |
| 29 | Liang 2016 | 2016 | Oxiracetam + nimodipine vs nimodipine | Racemic oxiracetam injection | 4 g/day | Once daily | IV infusion | Postoperative/acute care; exact injury-to-start time NR | 2 weeks | End of treatment (2 weeks) | Short-course IV (≤21 days) | Full-text source report |
| 28 | Si Daolin 2015 | 2015 | Oxiracetam + nimodipine vs nimodipine | Racemic oxiracetam injection | 4 g/day | Once daily | IV infusion | After surgery; preoperative disease duration 0.5–12 h | 14 days | Days 3, 7, and 14; day 14 used | Short-course IV (≤21 days) | Full-text source report |
| LOCATE | Liu 2025 (LOCATE) | 2025 | L-oxiracetam vs racemic oxiracetam vs placebo | L-oxiracetam injection; racemic oxiracetam injection; matched placebos | L-oxiracetam 4 g/day; racemic oxiracetam 6 g/day | Daily IV infusion | IV infusion | Typically within 72 h of injury | 14 days | Days 14, 30, 60, and 90 depending on outcome; primary LOTCA day 90 | L-enantiomer, phase 3, 14-day IV, 90-day primary outcome; synthesized separately | Liu et al. (2025), Tables 1–3 |
