# Supplementary material for: Efficacy and safety of oxiracetam for cognitive and functional outcomes after acute traumatic brain injury in Chinese populations: a systematic review and meta-analysis of randomized controlled trials
Source: Front Pharmacol. 2026 Sep 21;17:1894256. doi: 10.3389/fphar.2026.1894256 (PMC13635107; doi:10.3389/fphar.2026.1894256)
Supplement: Supplementary file 6 [file Table6.docx]

**Supplementary Table S6A**

**Source-linked extraction and verification of continuous outcomes**

Values are mean (SD) unless otherwise indicated. Source notation, baseline and endpoint values, assessment times, and source locations are reported for auditability. No SE-to-SD conversion was applied. Unnamed instruments and implausible dispersion values were excluded from primary analyses rather than assigned, corrected, or imputed. Duan (2013) reported an endpoint SD of 3.34; use of this source-verified value yielded the same pooled oxiracetam-versus-piracetam MMSE estimate at the reported precision.

| **Study / exact outcome** | **Comparison** | **Experimental arm** | **Control arm** | **Change values** | **Assessment time** | **Exact source notation** | **Source page/table** | **Conversion** | **Primary decision / audit note** |
| --- | --- | --- | --- | --- | --- | --- | --- | --- | --- |
| 01 Qi 2022 Mini-Mental State Examination (MMSE; 0–30; higher is better) | Oxiracetam add-on/balanced background therapy vs control | Oxiracetam + background therapy; n=75; baseline=12.11 (2.01); endpoint=26.84 (4.03) | Control/background therapy; n=75; baseline=12.23 (2.05); endpoint=17.9 (2.81) | NR unless separately reported; endpoint values analyzed | Day 14 | x̄ ± s (source defines as mean ± standard deviation) | PDF p.3, Table 2 | None | Included in primary analysis |
| 04 Cheng 2020 Mini-Mental State Examination (MMSE; 0–30; higher is better) | Oxiracetam add-on/balanced background therapy vs control | Oxiracetam + background therapy; n=50; baseline=13.48 (3.72); endpoint=24.65 (2.52) | Control/background therapy; n=50; baseline=14.04 (4.05); endpoint=22.61 (2.26) | NR unless separately reported; endpoint values analyzed | End of treatment (12 weeks) | x̄ ± s (source defines as mean ± standard deviation) | PDF p.2, Table 2 | None | Included in primary analysis |
| 08 Wang Xiangyang 2018 Mini-Mental State Examination (MMSE; 0–30; higher is better) | Oxiracetam add-on/balanced background therapy vs control | Oxiracetam + background therapy; n=24; baseline=17.92 (0.88); endpoint=25.63 (1.74) | Control/background therapy; n=24; baseline=17.83 (0.98); endpoint=22.65 (1.23) | NR unless separately reported; endpoint values analyzed | Days 7 and 14; day 14 used | x̄ ± s (source defines as mean ± standard deviation) | PDF p.3, Tables 1–2 | None | Included in primary analysis |
| 09 Fan 2016 Mini-Mental State Examination (MMSE; 0–30; higher is better) | Oxiracetam add-on/balanced background therapy vs control | Oxiracetam + background therapy; n=34; baseline=15.16 (3.31); endpoint=28.63 (1.62) | Control/background therapy; n=34; baseline=16.42 (5.62); endpoint=23.41 (5.58) | NR unless separately reported; endpoint values analyzed | End of treatment (3 weeks) | x̄ ± s (source defines as mean ± standard deviation) | PDF p.1, Tables 1–2 | None | Included in primary analysis |
| 10 Guo Xiaoming 2016 Mini-Mental State Examination (MMSE; 0–30; higher is better) | Oxiracetam add-on/balanced background therapy vs control | Oxiracetam + background therapy; n=55; baseline=20.63 (2.52); endpoint=29.25 (3.75) | Control/background therapy; n=55; baseline=20.58 (2.34); endpoint=23.01 (2.86) | NR unless separately reported; endpoint values analyzed | End of treatment (2 weeks) | x̄ ± s (source defines as mean ± standard deviation) | PDF p.2, Table 2 | None | Included in primary analysis |
| 11 Bai 2015 Mental-status scale (unnamed in source; 0–30; higher is better) | Oxiracetam add-on/balanced background therapy vs control | Oxiracetam + background therapy; n=37; baseline=15.1 (2.2); endpoint=26.8 (1.5) | Control/background therapy; n=35; baseline=14.6 (2.4); endpoint=22.1 (2.8) | NR unless separately reported; endpoint values analyzed | 15-day follow-up after treatment | x̄ ± s (source defines as mean ± standard deviation) | PDF p.1, Table 1 | None | Excluded from primary; sensitivity analysis only; Scale name was not stated; score range alone was not used to assign MMSE or NIHSS. |
| 16 Wu Jianlun 2014 Mini-Mental State Examination (MMSE; 0–30; higher is better) | Oxiracetam add-on/balanced background therapy vs control | Oxiracetam + background therapy; n=40; baseline=13.37 (2.38); endpoint=24.56 (3.35) | Control/background therapy; n=40; baseline=13.26 (2.16); endpoint=19.78 (2.67) | NR unless separately reported; endpoint values analyzed | End of treatment (3 weeks) | x̄ ± s (source defines as mean ± standard deviation) | PDF p.1, Table 1 | None | Included in primary analysis |
| 33 Yin 2014 Mini-Mental State Examination (MMSE; 0–30; higher is better) | Oxiracetam add-on/balanced background therapy vs control | Oxiracetam + background therapy; n=18; baseline=12.53 (2.86); endpoint=22.67 (4.02) | Control/background therapy; n=17; baseline=12.45 (2.81); endpoint=18.23 (3.12) | NR unless separately reported; endpoint values analyzed | After individualized treatment (2–3 weeks) | x̄ ± s (source defines as mean ± standard deviation) | PDF p.2, Table 1 | None | Included in primary analysis |
| 20 Chen Xuehua 2012 Mini-Mental State Examination (MMSE; 0–30; higher is better) | Oxiracetam add-on/balanced background therapy vs control | Oxiracetam + background therapy; n=38; baseline=15.97 (5.22); endpoint=28.91 (1.03) | Control/background therapy; n=38; baseline=16.24 (5.86); endpoint=24.26 (5.01) | NR unless separately reported; endpoint values analyzed | End of treatment (3 weeks) | x̄ ± s (source defines as mean ± standard deviation) | PDF p.1, Tables 1–2 | None | Included in primary analysis |
| 21 Huang 2012 Mini-Mental State Examination (MMSE; 0–30; higher is better) | Oxiracetam add-on/balanced background therapy vs control | Oxiracetam + background therapy; n=40; baseline=NR; endpoint=21.1 (9.3) | Control/background therapy; n=40; baseline=NR; endpoint=17.3 (5.2) | NR unless separately reported; endpoint values analyzed | Week 6 | x̄ ± s (source defines as mean ± standard deviation) | PDF p.1, Table 1 | None | Included in the primary meta-analysis; the week-6 endpoint was used, and assessment-time heterogeneity is documented in Supplementary Table S5. |
| 26 Yu Yigang 2005 Mini-Mental State Examination (MMSE; 0–30; higher is better) | Oxiracetam add-on/balanced background therapy vs control | Oxiracetam + background therapy; n=27; baseline=13.52 (6.4); endpoint=19.86 (8.27) | Control/background therapy; n=24; baseline=13.1 (7.29); endpoint=17.33 (8.18) | NR unless separately reported; endpoint values analyzed | 1 month (source also states a 2-month observation course) | x̄ ± s (source defines as mean ± standard deviation) | PDF p.2, unnumbered outcome table | None | The source states a 2-month observation course but reports MMSE at 1 month; the 1-month endpoint was used and the timing discrepancy was documented. |
| 10 Guo Xiaoming 2016 Barthel Index (BI) (0–100; higher is better; exact version not reported); domain: functional independence in basic activities of daily living | Oxiracetam add-on/balanced background therapy vs control | Oxiracetam + background therapy; n=55; baseline=50.68 (6.03); endpoint=63.26 (8.25) | Control/background therapy; n=55; baseline=50.43 (5.82); endpoint=55.84 (7.27) | NR unless separately reported; endpoint values analyzed | End of treatment (2 weeks) | x̄ ± s (source defines as mean ± standard deviation) | PDF p.2, Table 2 | None | Included in primary analysis |
| 13 Guo Yongxiang 2015 Barthel Index (BI) (0–100; higher is better; exact version not reported); domain: functional independence in basic activities of daily living | Oxiracetam add-on/balanced background therapy vs control | Oxiracetam + background therapy; n=46; baseline=70.41 (10.65); endpoint=89.96 (15.13) | Control/background therapy; n=46; baseline=71.25 (10.96); endpoint=77.13 (12.61) | NR unless separately reported; endpoint values analyzed | End of treatment (2 weeks) | x̄ ± s (source defines as mean ± standard deviation) | PDF p.2, Table 2 | None | Included in primary analysis |
| 18 Zhou 2013 Barthel Index (BI) (0–100; higher is better; exact version not reported); domain: functional independence in basic activities of daily living | Oxiracetam add-on/balanced background therapy vs control | Oxiracetam + background therapy; n=48; baseline=48.28 (10.68); endpoint=75.18 (12.87) | Control/background therapy; n=48; baseline=49.98 (11.57); endpoint=56.62 (11.63) | NR unless separately reported; endpoint values analyzed | End of treatment (21 days) | x̄ ± s (source defines as mean ± standard deviation) | PDF p.1, Table 1 | None | Included in primary analysis |
| 21 Huang 2012 Barthel Index (BI) (0–100; higher is better; exact version not reported); domain: functional independence in basic activities of daily living | Oxiracetam add-on/balanced background therapy vs control | Oxiracetam + background therapy; n=40; baseline=NR; endpoint=97.1 (10.1) | Control/background therapy; n=40; baseline=NR; endpoint=83.9 (23.2) | NR unless separately reported; endpoint values analyzed | Week 6 | x̄ ± s (source defines as mean ± standard deviation) | PDF p.1, Table 1 | None | Included in the primary meta-analysis; the week-6 endpoint was used, and assessment-time heterogeneity is documented in Supplementary Table S5. |
| 01 Qi 2022 National Institutes of Health Stroke Scale (NIHSS; lower is better) | Oxiracetam add-on/balanced background therapy vs control | Oxiracetam + background therapy; n=75; baseline=19.36 (3.43); endpoint=14.02 (2.56) | Control/background therapy; n=75; baseline=19.74 (3.56); endpoint=16.55 (3.01) | NR unless separately reported; endpoint values analyzed | Day 14 | x̄ ± s (source defines as mean ± standard deviation) | PDF p.3, Table 2 | None | Included in primary analysis |
| 04 Cheng 2020 National Institutes of Health Stroke Scale (NIHSS; lower is better) | Oxiracetam add-on/balanced background therapy vs control | Oxiracetam + background therapy; n=50; baseline=19.45 (4.61); endpoint=14.25 (3.44) | Control/background therapy; n=50; baseline=19.8 (4.84); endpoint=16.87 (3.43) | NR unless separately reported; endpoint values analyzed | End of treatment (12 weeks) | x̄ ± s (source defines as mean ± standard deviation) | PDF p.2, Table 2 | None | Included in primary analysis |
| 09 Fan 2016 National Institutes of Health Stroke Scale (NIHSS; lower is better) | Oxiracetam add-on/balanced background therapy vs control | Oxiracetam + background therapy; n=34; baseline=21.27 (5.69); endpoint=11.28 (3.92) | Control/background therapy; n=34; baseline=21.17 (5.38); endpoint=14.62 (4.61) | NR unless separately reported; endpoint values analyzed | End of treatment (3 weeks) | x̄ ± s (source defines as mean ± standard deviation) | PDF p.1, Tables 1–2 | None | Included in primary analysis |
| 11 Bai 2015 Neurological-deficit scale (unnamed in source; 0–30; lower is better) | Oxiracetam add-on/balanced background therapy vs control | Oxiracetam + background therapy; n=37; baseline=25.2 (2.8); endpoint=14.1 (1.8) | Control/background therapy; n=35; baseline=24.8 (3.2); endpoint=18.3 (2.6) | NR unless separately reported; endpoint values analyzed | 15-day follow-up after treatment | x̄ ± s (source defines as mean ± standard deviation) | PDF p.1, Table 1 | None | Excluded from primary; sensitivity analysis only; Scale name was not stated; score range alone was not used to assign MMSE or NIHSS. |
| 16 Wu Jianlun 2014 National Institutes of Health Stroke Scale (NIHSS; lower is better) | Oxiracetam add-on/balanced background therapy vs control | Oxiracetam + background therapy; n=40; baseline=24.58 (4.68); endpoint=11.24 (2.6) | Control/background therapy; n=40; baseline=23.96 (5.22); endpoint=15.63 (3.41) | NR unless separately reported; endpoint values analyzed | End of treatment (3 weeks) | x̄ ± s (source defines as mean ± standard deviation) | PDF p.1, Table 1 | None | Included in primary analysis |
| 33 Yin 2014 National Institutes of Health Stroke Scale (NIHSS; lower is better) | Oxiracetam add-on/balanced background therapy vs control | Oxiracetam + background therapy; n=18; baseline=22.95 (7.11); endpoint=9.56 (3.23) | Control/background therapy; n=17; baseline=23.15 (7.23); endpoint=16.56 (4.56) | NR unless separately reported; endpoint values analyzed | After individualized treatment (2–3 weeks) | x̄ ± s (source defines as mean ± standard deviation) | PDF p.2, Table 1 | None | Included in primary analysis |
| 18 Zhou 2013 National Institutes of Health Stroke Scale (NIHSS; lower is better) | Oxiracetam add-on/balanced background therapy vs control | Oxiracetam + background therapy; n=48; baseline=24.81 (9.18); endpoint=13.82 (4.58) | Control/background therapy; n=48; baseline=25.18 (9.98); endpoint=15.58 (4.96) | NR unless separately reported; endpoint values analyzed | End of treatment (21 days) | x̄ ± s (source defines as mean ± standard deviation) | PDF p.1, Table 1 | None | Included in primary analysis |
| 20 Chen Xuehua 2012 National Institutes of Health Stroke Scale (NIHSS; lower is better) | Oxiracetam add-on/balanced background therapy vs control | Oxiracetam + background therapy; n=38; baseline=21.87 (6.99); endpoint=11.05 (5.21) | Control/background therapy; n=38; baseline=22.03 (7.28); endpoint=13.24 (4.22) | NR unless separately reported; endpoint values analyzed | End of treatment (3 weeks) | x̄ ± s (source defines as mean ± standard deviation) | PDF p.1, Tables 1–2 | None | Included in primary analysis |
| 21 Huang 2012 National Institutes of Health Stroke Scale (NIHSS; lower is better) | Oxiracetam add-on/balanced background therapy vs control | Oxiracetam + background therapy; n=40; baseline=NR; endpoint=4.3 (4.2) | Control/background therapy; n=40; baseline=NR; endpoint=7.5 (7.1) | NR unless separately reported; endpoint values analyzed | Week 6 | x̄ ± s (source defines as mean ± standard deviation) | PDF p.1, Table 1 | None | Included in the primary meta-analysis; the week-6 endpoint was used, and assessment-time heterogeneity is documented in Supplementary Table S5. |
| 01 Qi 2022 Glasgow Coma Scale (GCS; 3–15; higher is better) | Oxiracetam add-on/balanced background therapy vs control | Oxiracetam + background therapy; n=75; baseline=4.83 (0.37); endpoint=10.86 (0.94) | Control/background therapy; n=75; baseline=4.79 (0.34); endpoint=7.54 (0.86) | NR unless separately reported; endpoint values analyzed | Day 14 | x̄ ± s (source defines as mean ± standard deviation) | PDF p.3, Table 2 | None | Included in primary analysis |
| 02 Liu Haiyan 2020 Glasgow Coma Scale (GCS; 3–15; higher is better) | Oxiracetam add-on/balanced background therapy vs control | Oxiracetam + background therapy; n=48; baseline=4.74 (0.31); endpoint=10.41 (0.26) | Control/background therapy; n=48; baseline=4.79 (0.32); endpoint=8.16 (0.17) | NR unless separately reported; endpoint values analyzed | Admission; days 3, 7, and 14 | x̄ ± s (source defines as mean ± standard deviation) | PDF p.2, Table 1 | None | Included in the primary meta-analysis. The small endpoint SDs (0.26 and 0.17) were verified as printed under mean ± SD notation in PDF p.2, Table 1. |
| 03 Tan 2020 Glasgow Coma Scale (GCS; 3–15; higher is better) | Oxiracetam add-on/balanced background therapy vs control | Oxiracetam + background therapy; n=38; baseline=7.37 (1.18); endpoint=14.11 (0.84) | Control/background therapy; n=38; baseline=7.41 (1.21); endpoint=9.11 (1.46) | NR unless separately reported; endpoint values analyzed | End of 180-day treatment | x̄ ± s (source defines as mean ± standard deviation) | PDF p.2, Table 1 | None | Included in primary analysis; Oral 180-day regimen with balanced hyperbaric oxygen; clinically distinct from short-course IV studies. |
| 08 Wang Xiangyang 2018 Glasgow Coma Scale (GCS; 3–15; higher is better) | Oxiracetam add-on/balanced background therapy vs control | Oxiracetam + background therapy; n=24; baseline=9.38 (0.49); endpoint=13.5 (0.93) | Control/background therapy; n=24; baseline=9.47 (0.51); endpoint=12.43 (0.59) | NR unless separately reported; endpoint values analyzed | Days 7 and 14; day 14 used | x̄ ± s (source defines as mean ± standard deviation) | PDF p.3, Tables 1–2 | None | Included in primary analysis |
| 10 Guo Xiaoming 2016 Glasgow Coma Scale (GCS; 3–15; higher is better) | Oxiracetam add-on/balanced background therapy vs control | Oxiracetam + background therapy; n=55; baseline=6.3 (1.12); endpoint=10.96 (1.89) | Control/background therapy; n=55; baseline=6.24 (1.05); endpoint=8.73 (1.53) | NR unless separately reported; endpoint values analyzed | End of treatment (2 weeks) | x̄ ± s (source defines as mean ± standard deviation) | PDF p.2, Table 2 | None | Included in primary analysis |
| 13 Guo Yongxiang 2015 Glasgow Coma Scale (GCS; 3–15; higher is better) | Oxiracetam add-on/balanced background therapy vs control | Oxiracetam + background therapy; n=46; baseline=5.58 (1.25); endpoint=10.96 (2.31) | Control/background therapy; n=46; baseline=5.6 (1.28); endpoint=9.4 (2.03) | NR unless separately reported; endpoint values analyzed | End of treatment (2 weeks) | x̄ ± s (source defines as mean ± standard deviation) | PDF p.2, Table 2 | None | Included in primary analysis |
| 12 Zhai Zhongwen 2015 Glasgow Coma Scale (GCS; 3–15; higher is better) | Oxiracetam add-on/balanced background therapy vs control | Oxiracetam + background therapy; n=50; baseline=7.96 (3.63); day-21 endpoint=14.31 (2.25) | Control/background therapy; n=50; baseline=7.56 (2.54); day-21 endpoint=12.26 (11.17) | NR unless separately reported; endpoint values analyzed | Day 21 (source also reports days 7 and 14) | x̄ ± s (source defines as mean ± standard deviation) | PDF p.2, Table 2 | None; value excluded | Excluded from the primary GCS synthesis because the day-21 control SD of 11.17 is incompatible with the GCS range. No decimal-place correction or imputation was used. |
| 16 Wu Jianlun 2014 Glasgow Coma Scale (GCS; 3–15; higher is better) | Oxiracetam add-on/balanced background therapy vs control | Oxiracetam + background therapy; n=40; baseline=8.34 (1.13); endpoint=14.78 (2.32) | Control/background therapy; n=40; baseline=8.41 (1.09); endpoint=11.23 (2.45) | NR unless separately reported; endpoint values analyzed | End of treatment (3 weeks) | x̄ ± s (source defines as mean ± standard deviation) | PDF p.1, Table 1 | None | Included in primary analysis |
| 33 Yin 2014 Glasgow Coma Scale (GCS; 3–15; higher is better) | Oxiracetam add-on/balanced background therapy vs control | Oxiracetam + background therapy; n=18; baseline=9.56 (2.71); endpoint=14.65 (2.53) | Control/background therapy; n=17; baseline=9.46 (2.85); endpoint=10.58 (2.13) | NR unless separately reported; endpoint values analyzed | After individualized treatment (2–3 weeks) | x̄ ± s (source defines as mean ± standard deviation) | PDF p.2, Table 1 | None | Included in primary analysis |
| 18 Zhou 2013 Glasgow Coma Scale (GCS; 3–15; higher is better) | Oxiracetam add-on/balanced background therapy vs control | Oxiracetam + background therapy; n=48; baseline=9.51 (1.91); endpoint=14.02 (1.86) | Control/background therapy; n=48; baseline=9.86 (1.68); endpoint=12.81 (1.63) | NR unless separately reported; endpoint values analyzed | End of treatment (21 days) | x̄ ± s (source defines as mean ± standard deviation) | PDF p.1, Table 1 | None | Included in primary analysis |
| 07 Liu Min 2018 Mini-Mental State Examination (MMSE; 0–30; higher is better) | Oxiracetam vs piracetam | Oxiracetam; n=40; baseline=12.32 (2.79); endpoint=19.83 (4.32) | Piracetam; n=40; baseline=12.33 (2.78); endpoint=17.19 (5.23) | NR unless separately reported; endpoint values analyzed | After treatment; exact time NR | x̄ ± s (source defines as mean ± standard deviation) | PDF p.1, Table 1 | None | Included in primary analysis |
| 14 Chen Xu 2014 Mini-Mental State Examination (MMSE; 0–30; higher is better) | Oxiracetam vs piracetam | Oxiracetam; n=26; baseline=13.12 (1.51); endpoint=24.23 (1.92) | Piracetam; n=26; baseline=13.35 (1.32); endpoint=19.92 (1.79) | NR unless separately reported; endpoint values analyzed | End of treatment (3 weeks) | Means ± SD | PDF p.2, Table 1 | None | Included in primary analysis |
| 15 Liu Bin 2014 Mini-Mental State Examination (MMSE; 0–30; higher is better) | Oxiracetam vs piracetam | Oxiracetam; n=25; baseline=15.48 (3.72); endpoint=28.01 (4.35) | Piracetam; n=25; baseline=15.47 (3.86); endpoint=23.86 (4.66) | NR unless separately reported; endpoint values analyzed | End of treatment (21 days) | x̄ ± s (source defines as mean ± standard deviation) | PDF p.1, Table 1 | None | Included in primary analysis |
| 17 Duan 2013 Mini-Mental State Examination (MMSE; 0–30; higher is better) | Oxiracetam vs piracetam | Oxiracetam; n=48; baseline=12.72 (3.36); endpoint=21.59 (3.34) | Piracetam; n=47; baseline=12.68 (3.35); endpoint=17.36 (3.46) | NR unless separately reported; endpoint values analyzed | End of 3 courses (3 weeks) | x̄ ± s (source defines as mean ± standard deviation) | PDF p.2, Table 1 | None | The source report gives an endpoint SD of 3.34; this source-reported value was used. The pooled active-comparator MMSE estimate was MD 4.05 (95% CI 3.51–4.60; I² = 0%). |
| 23 Liu Zhongyuan 2012 Mini-Mental State Examination (MMSE; 0–30; higher is better) | Oxiracetam vs piracetam | Oxiracetam; n=60; baseline=13.21 (3.73); endpoint=22.19 (4.71) | Piracetam; n=60; baseline=13.02 (3.08); endpoint=18.83 (4.98) | NR unless separately reported; endpoint values analyzed | 3–6-month follow-up (mean 4.46 months) | x̄ ± s (source defines as mean ± standard deviation) | PDF p.2, Table 1 | None | Included in primary analysis; Endpoint appears to be the 3–6-month follow-up (mean 4.46 months), not the 2-week treatment end. |
| 22 Sun Xiuhong 2012 Mini-Mental State Examination (MMSE; 0–30; higher is better) | Oxiracetam vs piracetam | Oxiracetam; n=48; baseline=12.75 (2.07); endpoint=19.77 (5.02) | Piracetam; n=48; baseline=12.26 (2.82); endpoint=17.21 (5.33) | NR unless separately reported; endpoint values analyzed | Week 3 | x̄ ± s (source defines as mean ± standard deviation) | PDF p.1, Tables 1–2 | None | Included in primary analysis |
| 24 Guo Xiaofeng 2009 Mini-Mental State Examination (MMSE; 0–30; higher is better) | Oxiracetam vs piracetam | Oxiracetam; n=63; baseline=12.75 (2.08); endpoint=21.79 (5.04) | Piracetam; n=63; baseline=12.16 (2.81); endpoint=17.21 (5.32) | NR unless separately reported; endpoint values analyzed | Day 21 | x̄ ± s (source defines as mean ± standard deviation) | PDF p.2, Table 1 | None | Included in primary analysis |
| 27 Shu Kai 2005 Mini-Mental State Examination (MMSE; 0–30; higher is better) | Oxiracetam vs piracetam | Oxiracetam; n=60; baseline=16.43 (4.62); endpoint=28.84 (1.98) | Piracetam; n=60; baseline=16.84 (3.51); endpoint=24.14 (4.75) | NR unless separately reported; endpoint values analyzed | End of treatment (30 days) | x̄ ± s (source defines as mean ± standard deviation) | PDF p.2, Table 2 | None | Included in primary analysis |
| 15 Liu Bin 2014 Barthel Index (BI) (0–100; higher is better; exact version not reported); domain: functional independence in basic activities of daily living | Oxiracetam vs piracetam | Oxiracetam; n=25; baseline=48.81 (8.03); endpoint=76.42 (11.87) | Piracetam; n=25; baseline=48.64 (8.21); endpoint=55.24 (10.28) | NR unless separately reported; endpoint values analyzed | End of treatment (21 days) | x̄ ± s (source defines as mean ± standard deviation) | PDF p.1, Table 1 | None | Included in primary analysis |
| 07 Liu Min 2018 National Institutes of Health Stroke Scale (NIHSS; lower is better) | Oxiracetam vs piracetam | Oxiracetam; n=40; baseline=22.82 (7.92); endpoint=16.54 (4.01) | Piracetam; n=40; baseline=22.79 (7.89); endpoint=18.93 (4.23) | NR unless separately reported; endpoint values analyzed | After treatment; exact time NR | x̄ ± s (source defines as mean ± standard deviation) | PDF p.1, Table 1 | None | Included in primary analysis |
| 14 Chen Xu 2014 National Institutes of Health Stroke Scale (NIHSS; lower is better) | Oxiracetam vs piracetam | Oxiracetam; n=26; baseline=24.15 (2.07); endpoint=10.73 (1.31) | Piracetam; n=26; baseline=23.62 (2.32); endpoint=15.12 (1.77) | NR unless separately reported; endpoint values analyzed | End of treatment (3 weeks) | Means ± SD | PDF p.2, Table 1 | None | Included in primary analysis |
| 15 Liu Bin 2014 National Institutes of Health Stroke Scale (NIHSS; lower is better) | Oxiracetam vs piracetam | Oxiracetam; n=25; baseline=24.85 (4.81); endpoint=12.18 (2.01) | Piracetam; n=25; baseline=24.78 (4.74); endpoint=15.92 (2.62) | NR unless separately reported; endpoint values analyzed | End of treatment (21 days) | x̄ ± s (source defines as mean ± standard deviation) | PDF p.1, Table 1 | None | Included in primary analysis |
| 17 Duan 2013 National Institutes of Health Stroke Scale (NIHSS; lower is better) | Oxiracetam vs piracetam | Oxiracetam; n=48; baseline=22.75 (6.38); endpoint=10.71 (3.19) | Piracetam; n=47; baseline=22.78 (6.37); endpoint=15.89 (5.78) | NR unless separately reported; endpoint values analyzed | End of 3 courses (3 weeks) | x̄ ± s (source defines as mean ± standard deviation) | PDF p.2, Table 1 | None | Included in primary analysis |
| 19 Yang 2013 National Institutes of Health Stroke Scale (NIHSS; lower is better) | Oxiracetam vs piracetam | Oxiracetam; n=43; baseline=25.91 (4.71); endpoint=14.37 (7.92) | Piracetam; n=43; baseline=26.04 (5.17); endpoint=19.16 (5.17) | NR unless separately reported; endpoint values analyzed | End of treatment (21 days) | x̄ ± s (source defines as mean ± standard deviation) | PDF p.2, Results text | None | Included in primary analysis |
| 23 Liu Zhongyuan 2012 National Institutes of Health Stroke Scale (NIHSS; lower is better) | Oxiracetam vs piracetam | Oxiracetam; n=60; baseline=23.53 (5.43); endpoint=11.38 (2.48) | Piracetam; n=60; baseline=24.12 (5.6); endpoint=14.43 (2.78) | NR unless separately reported; endpoint values analyzed | 3–6-month follow-up (mean 4.46 months) | x̄ ± s (source defines as mean ± standard deviation) | PDF p.2, Table 1 | None | Included in primary analysis; Endpoint appears to be the 3–6-month follow-up (mean 4.46 months), not the 2-week treatment end. |
| 22 Sun Xiuhong 2012 National Institutes of Health Stroke Scale (NIHSS; lower is better) | Oxiracetam vs piracetam | Oxiracetam; n=48; baseline=22.54 (8.46); endpoint=16.57 (4.11) | Piracetam; n=48; baseline=22.76 (8.49); endpoint=18.78 (4.99) | NR unless separately reported; endpoint values analyzed | Week 3 | x̄ ± s (source defines as mean ± standard deviation) | PDF p.1, Tables 1–2 | None | Included in primary analysis |
| 24 Guo Xiaofeng 2009 National Institutes of Health Stroke Scale (NIHSS; lower is better) | Oxiracetam vs piracetam | Oxiracetam; n=63; baseline=22.53 (8.44); endpoint=10.15 (2.97) | Piracetam; n=63; baseline=22.75 (8.48); endpoint=15.76 (4.97) | NR unless separately reported; endpoint values analyzed | Day 21 | x̄ ± s (source defines as mean ± standard deviation) | PDF p.2, Table 1 | None | Included in primary analysis |
| 25 Zhang Guoqing 2008 National Institutes of Health Stroke Scale (NIHSS; lower is better) | Oxiracetam vs piracetam | Oxiracetam; n=27; baseline=22.95 (9.14); endpoint=11.17 (4.53) | Piracetam; n=27; baseline=21.48 (10.14); endpoint=12.53 (4.53) | NR unless separately reported; endpoint values analyzed | Day 21 | x̄ ± s (source defines as mean ± standard deviation) | PDF p.2, Results text | None | Included in primary analysis |
| 07 Liu Min 2018 Glasgow Coma Scale (GCS; 3–15; higher is better) | Oxiracetam vs piracetam | Oxiracetam; n=40; baseline=8.9 (2.18); endpoint=13.09 (3.24) | Piracetam; n=40; baseline=8.89 (2.19); endpoint=10.79 (2.89) | NR unless separately reported; endpoint values analyzed | After treatment; exact time NR | x̄ ± s (source defines as mean ± standard deviation) | PDF p.1, Table 1 | None | Included in primary analysis |
| 14 Chen Xu 2014 Glasgow Coma Scale (GCS; 3–15; higher is better) | Oxiracetam vs piracetam | Oxiracetam; n=26; baseline=8.19 (0.71); endpoint=14.58 (1.39) | Piracetam; n=26; baseline=8.12 (0.71); endpoint=10.92 (1.35) | NR unless separately reported; endpoint values analyzed | End of treatment (3 weeks) | Means ± SD | PDF p.2, Table 1 | None | Included in primary analysis |
| 17 Duan 2013 Glasgow Coma Scale (GCS; 3–15; higher is better) | Oxiracetam vs piracetam | Oxiracetam; n=48; baseline=10.09 (0.45); endpoint=13.65 (0.55) | Piracetam; n=47; baseline=10.07 (0.45); endpoint=12.33 (0.54) | NR unless separately reported; endpoint values analyzed | End of 3 courses (3 weeks) | x̄ ± s (source defines as mean ± standard deviation) | PDF p.2, Table 1 | None | Included in primary analysis |
| 19 Yang 2013 Glasgow Coma Scale (GCS; 3–15; higher is better) | Oxiracetam vs piracetam | Oxiracetam; n=43; baseline=6 (1.66); endpoint=10.81 (2.37) | Piracetam; n=43; baseline=6.09 (1.62); endpoint=9.78 (2.52) | NR unless separately reported; endpoint values analyzed | End of treatment (21 days) | x̄ ± s (source defines as mean ± standard deviation) | PDF p.2, Results text | None | Included in primary analysis |
| 23 Liu Zhongyuan 2012 Glasgow Coma Scale (GCS; 3–15; higher is better) | Oxiracetam vs piracetam | Oxiracetam; n=60; baseline=8.46 (1.22); endpoint=14.86 (2.29) | Piracetam; n=60; baseline=8.51 (1.23); endpoint=11.18 (2.31) | NR unless separately reported; endpoint values analyzed | 3–6-month follow-up (mean 4.46 months) | x̄ ± s (source defines as mean ± standard deviation) | PDF p.2, Table 1 | None | Included in primary analysis; Endpoint appears to be the 3–6-month follow-up (mean 4.46 months), not the 2-week treatment end. |
| 22 Sun Xiuhong 2012 Glasgow Coma Scale (GCS; 3–15; higher is better) | Oxiracetam vs piracetam | Oxiracetam; n=48; baseline=8.79 (2.14); endpoint=13.12 (3.35) | Piracetam; n=48; baseline=8.83 (2.25); endpoint=11.74 (2.87) | NR unless separately reported; endpoint values analyzed | Day 14 | x̄ ± s (source defines as mean ± standard deviation) | PDF p.1, Tables 1–2 | None | Included in primary analysis |
| 24 Guo Xiaofeng 2009 Glasgow Coma Scale (GCS; 3–15; higher is better) | Oxiracetam vs piracetam | Oxiracetam; n=63; baseline=9.65 (2.11); endpoint=14.15 (2.29) | Piracetam; n=63; baseline=9.58 (2.34); endpoint=13.59 (2.43) | NR unless separately reported; endpoint values analyzed | Day 21 | x̄ ± s (source defines as mean ± standard deviation) | PDF p.2, Table 1 | None | Included in primary analysis |
| 27 Shu Kai 2005 Glasgow Coma Scale (GCS; 3–15; higher is better) | Oxiracetam vs piracetam | Oxiracetam; n=60; baseline=8.43 (3.41); endpoint=14.84 (3.09) | Piracetam; n=60; baseline=8.84 (3.44); endpoint=12.14 (2.63) | NR unless separately reported; endpoint values analyzed | End of treatment (30 days) | x̄ ± s (source defines as mean ± standard deviation) | PDF p.2, Table 2 | None | Included in primary analysis |
| 32 Yun Feifei 2021 National Institutes of Health Stroke Scale (NIHSS; lower is better) | Oxiracetam plus nimodipine vs nimodipine | Oxiracetam + nimodipine; n=50; baseline=28.64 (3.46); endpoint=1.58 (1.25) | Nimodipine; n=50; baseline=28.57 (3.48); endpoint=5.52 (3.18) | NR unless separately reported; endpoint values analyzed | End of treatment (2 weeks) | x̄ ± s (source defines as mean ± standard deviation) | PDF p.2, Results text | None | Included in primary analysis |
| 30 Zhang Lin 2017 National Institutes of Health Stroke Scale (NIHSS; lower is better) | Oxiracetam plus nimodipine vs nimodipine | Oxiracetam + nimodipine; n=30; baseline=217.46 (3.48); endpoint=1.52 (1.48) | Nimodipine; n=30; baseline=27.91 (3.98); endpoint=5.48 (3.26) | NR unless separately reported; endpoint values analyzed | End of treatment (2 weeks) | x̄ ± s (source defines as mean ± standard deviation) | PDF p.2, Table 2 | None | Included in primary analysis; Source prints experimental baseline NIHSS as 217.46, outside the scale range; not corrected and not used in endpoint-value meta-analysis. |
| 29 Liang 2016 National Institutes of Health Stroke Scale (NIHSS; lower is better) | Oxiracetam plus nimodipine vs nimodipine | Oxiracetam + nimodipine; n=38; baseline=27.84 (3.49); endpoint=1.56 (1.22) | Nimodipine; n=37; baseline=28.04 (3.94); endpoint=5.69 (3.44) | NR unless separately reported; endpoint values analyzed | End of treatment (2 weeks) | x̄ ± s (source defines as mean ± standard deviation) | PDF p.2, Table 2 | None | Included in primary analysis |
| 28 Si Daolin 2015 National Institutes of Health Stroke Scale (NIHSS; lower is better) | Oxiracetam plus nimodipine vs nimodipine | Oxiracetam + nimodipine; n=62; baseline=27.05 (4.37); endpoint=1.55 (1.5) | Nimodipine; n=68; baseline=28.81 (3.61); endpoint=5.57 (3.28) | NR unless separately reported; endpoint values analyzed | Days 3, 7, and 14; day 14 used | x̄ ± s (source defines as mean ± standard deviation) | PDF p.3, Tables 2–3 | None | Included in primary analysis |
| 31 Sun Gang 2018 Glasgow Coma Scale (GCS; 3–15; higher is better) | Oxiracetam plus nimodipine vs nimodipine | Oxiracetam + nimodipine; n=50; baseline=NR; endpoint=14.56 (1.35) | Nimodipine; n=50; baseline=NR; endpoint=6.39 (1.47) | NR unless separately reported; endpoint values analyzed | End of treatment (14 days) | x̄ ± s (source defines as mean ± standard deviation) | PDF p.1, Results text | None | Included in primary analysis |
| 28 Si Daolin 2015 Glasgow Coma Scale (GCS; 3–15; higher is better) | Oxiracetam plus nimodipine vs nimodipine | Oxiracetam + nimodipine; n=62; baseline=6.02 (1.49); endpoint=14.53 (0.7) | Nimodipine; n=68; baseline=6.37 (1.56); endpoint=13.41 (0.78) | NR unless separately reported; endpoint values analyzed | Days 3, 7, and 14; day 14 used | x̄ ± s (source defines as mean ± standard deviation) | PDF p.3, Tables 2–3 | None | Included in primary analysis |
