# Supplementary material for: Efficacy and safety of oxiracetam for cognitive and functional outcomes after acute traumatic brain injury in Chinese populations: a systematic review and meta-analysis of randomized controlled trials
Source: Front Pharmacol. 2026 Sep 21;17:1894256. doi: 10.3389/fphar.2026.1894256 (PMC13635107; doi:10.3389/fphar.2026.1894256)
Supplement: Supplementary file 7 [file Table7.docx]

**Supplementary Table S6B. Source-linked extraction of dichotomous efficacy and safety outcomes**

Arm-level event counts are shown only when explicitly reported or directly derivable; NR indicates not reported. Values with unreported arm-specific counts or unresolved internal inconsistencies were excluded from quantitative synthesis. Safety terminology follows the source reports because surveillance methods and definitions varied. The strict add-on or balanced-background safety synthesis included five source-verifiable reports (n = 504; RR 1.14, 95% CI 0.64–2.04; I² = 0%).

| **Study** | **Comparison** | **Outcome** | **Assessment time** | **Experimental arm (events/total)** | **Control arm (events/total)** | **Source statistic and location** | **Analysis status** | **Verification / notes** |
| --- | --- | --- | --- | --- | --- | --- | --- | --- |
| Qi 2022 | Oxiracetam add-on / balanced background vs control | Investigator-defined response | Day 14 / end of 14-day treatment | Oxiracetam + background therapy; 65/75 | Control/background therapy; 45/75 | PDF p.3, Results 2.1, Table 1 | Included in the primary meta-analysis | Counts verified against the source table. |
| Liu Haiyan 2020 | Oxiracetam add-on / balanced background vs control | Investigator-defined response | 6-month follow-up | Oxiracetam + background therapy; 41/48 | Control/background therapy; 33/48 | PDF p.1, Methods (GOS definition); p.2, Results subsection 3 | Included in the primary meta-analysis | Response was based on the source-defined GOS criterion. |
| Zhai Jingguang 2018 | Oxiracetam add-on / balanced background vs control | Investigator-defined response | Day 14 / end of treatment | Oxiracetam + background therapy; 49/55 | Control/background therapy; 37/55 | PDF p.2, Results subsection 1, Table 1 | Included in the primary meta-analysis | Counts verified against the source table. |
| Guo Xiaoming 2016 | Oxiracetam add-on / balanced background vs control | Investigator-defined response | 2 weeks / end of treatment | Oxiracetam + background therapy; 52/55 | Control/background therapy; 43/55 | PDF p.2, Results 2.1, Table 1 | Included in the primary meta-analysis | Counts verified against the source table. |
| Guo Yongxiang 2015 | Oxiracetam add-on / balanced background vs control | Investigator-defined response | 2 weeks / end of treatment | Oxiracetam + background therapy; 44/46 | Control/background therapy; 37/46 | PDF p.2, Results 2.1 | Included in the primary meta-analysis | Counts were derived exactly from the reported percentages (95.65% and 80.44%). |
| Qi 2022 | Oxiracetam add-on / balanced background vs control | Reported complications/adverse-event-type outcomes | During the 14-day treatment | Oxiracetam + background therapy; 11/75 | Control/background therapy; 10/75 | PDF p.3, Results 2.4, Table 4 | Included in the primary safety meta-analysis | The source labels these outcomes as complications; definition heterogeneity is acknowledged. |
| Liu Haiyan 2020 | Oxiracetam add-on / balanced background vs control | Reported adverse reactions | During the 14-day treatment | Oxiracetam + background therapy; 3/48 | Control/background therapy; 2/48 | PDF p.2, Results subsection 2 | Included in the primary safety meta-analysis | Oxiracetam: pruritus 2, WBC decrease 1; control: rash 1, WBC decrease 1. |
| Tan 2020 | Oxiracetam add-on / balanced background vs control | Reported complications/adverse-event-type outcomes | Over three 60-day courses (approximately 180 days) | Oxiracetam + background therapy; 2/38 | Control/background therapy; 3/38 | PDF p.1, Methods 1.2 and Results 2.3, Table 3 | Included in the primary safety meta-analysis | Events were intracranial-pressure increase, intracranial hemorrhage, or traumatic epilepsy; source definition differs from conventional drug-related AE reporting. |
| Guo Xiaoming 2016 | Oxiracetam add-on / balanced background vs control | Reported adverse events/adverse reactions | During the 2-week treatment | Oxiracetam + background therapy; 5/55 | Control/background therapy; 4/55 | PDF p.2, Results 2.4, Table 4 | Included in the primary safety meta-analysis | Arm-specific counts verified. |
| Bai 2015 | Oxiracetam add-on / balanced background vs control | Reported adverse reactions | At the end of the 20-day treatment | Oxiracetam + background therapy; 1/37 | Control/background therapy; 0/35 | PDF p.1, Methods 1.2 and Results 2.2 | Included in the primary safety meta-analysis | The source explicitly states no adverse reaction in the control arm. |
| Zhai Zhongwen 2015 | Oxiracetam add-on / balanced background vs control | Reported adverse reactions | 3 months after treatment | Oxiracetam + background therapy; 1/50 | Control/background therapy; NR/50 | PDF p.2, Methods 1.4 and Results 2.4 | Not quantitatively estimable; narrative only | The control-arm event count was not reported; no zero-event assumption was made. |
| Wu Jianlun 2014 | Oxiracetam add-on / balanced background vs control | Reported adverse reactions | At the end of the 3-week treatment | Oxiracetam + background therapy; 2/40 | Control/background therapy; NR/40 | PDF p.1, Results 2.2 | Excluded from quantitative synthesis; narrative only | The source reports two rashes in the oxiracetam arm but does not explicitly report the control-arm count. |
| Liu Haiyan 2020 | Oxiracetam add-on / balanced background vs control | All-cause mortality | 12-month follow-up | Oxiracetam + background therapy; 1/48 | Control/background therapy; 7/48 | PDF p.2, Results subsection 3 | Narrative only; not classified as a treatment-related adverse event | All-cause mortality during follow-up. |
| Sui 2019 | Oxiracetam add-on / balanced background vs control | All-cause mortality | Monthly follow-up during March 2015–November 2017; individual duration varied | Oxiracetam + background therapy; 3/40 | Control/background therapy; 14/40 | PDF p.2, Methods 1.2.3; p.3, Results 2.3 | Narrative only; not classified as a treatment-related adverse event | Follow-up duration was not a uniform fixed endpoint. |
| Shu Kai 2005 | Oxiracetam vs piracetam | Investigator-defined response | Day 30 / end of treatment | Oxiracetam; 50/60 | Piracetam; 43/60 | PDF p.2, Table 3 | Single-study comparator-specific analysis | Responder counts were derived from the source categories (20 + 30 vs 12 + 31). |
| Duan 2013 | Oxiracetam vs piracetam | Reported adverse reactions | During three 1-week courses (3 weeks total) | Oxiracetam; 1/48 | Piracetam; 6/47 | PDF p.2, Methods 1.2 and Results 2.2 | Single-study comparator-specific analysis | Oxiracetam: dizziness 1; piracetam: nausea 1, abdominal discomfort 2, mild dizziness 3. |
| Yun Feifei 2021 | Oxiracetam + nimodipine vs nimodipine | Investigator-defined response | 2 weeks / end of treatment | Oxiracetam + nimodipine; 47/50 | Nimodipine; 35/50 | PDF p.2, Methods 1.2 and Results 2.1 | Included in the primary meta-analysis | Counts verified. |
| Sun Gang 2018 | Oxiracetam + nimodipine vs nimodipine | Investigator-defined response | Day 14 / end of treatment | Oxiracetam + nimodipine; 49/50 | Nimodipine; 39/50 | PDF p.1, Methods 1.2 and Results 2.1 | Included in the primary meta-analysis | Counts verified. |
| Zhang Lin 2017 | Oxiracetam + nimodipine vs nimodipine | Investigator-defined response | 2 weeks / end of treatment | Oxiracetam + nimodipine; 27/30 | Nimodipine; NR/30 | PDF p.1, Results text; p.2, Table 1 | Excluded from quantitative synthesis | The reported 73.3% implies 22/30, whereas the category counts sum to 21/30; the source inconsistency was unresolved. |
| Liang 2016 | Oxiracetam + nimodipine vs nimodipine | Investigator-defined response | 2 weeks / end of treatment | Oxiracetam + nimodipine; 33/38 | Nimodipine; 25/37 | PDF p.2, Methods 1.3 and Results 2.1, Table 1 | Included in the primary meta-analysis | Counts verified. |
| Si Daolin 2015 | Oxiracetam + nimodipine vs nimodipine | Investigator-defined response | Day 14 / end of treatment | Oxiracetam + nimodipine; 50/62 | Nimodipine; 42/68 | PDF p.3, Results 2.1, Table 1 | Included in the primary meta-analysis | Counts verified. |
| Fan 2016 | Oxiracetam add-on / balanced background vs control | Narrative adverse-reaction statement | After the 3-week treatment; observation window not separately specified | Oxiracetam + background therapy; NR/34 | Control/background therapy; NR/34 | PDF p.1, discussion/concluding paragraph | Narrative only; no arm-level event counts | The source states that no obvious adverse reactions occurred but does not provide group-specific counts; it was not coded as a double-zero report. |
| Chen Xuehua 2012 | Oxiracetam add-on / balanced background vs control | Reported adverse reactions | During the 3-week treatment | Oxiracetam + background therapy; 0/38 | Control/background therapy; 0/38 | PDF p.1, Results 2.3 continuing on p.2 | Narrative double-zero report; not estimable as a log RR | Both arms explicitly reported zero adverse reactions. |
| Guo Xiaofeng 2009 | Oxiracetam vs piracetam | Reported adverse drug reactions | Through Day 21 / end of treatment | Oxiracetam; 0/63 | Piracetam; 0/63 | PDF p.2, Results 2.3 | Narrative double-zero report; not estimable as a log RR | Zero values apply to adverse drug reactions; one mild seizure in the piracetam arm was judged unrelated to treatment by the source authors. |
| Shu Kai 2005 | Oxiracetam vs piracetam | Reported adverse reactions | Day 30 / end of treatment | Oxiracetam; 0/60 | Piracetam; 0/60 | PDF p.2, Results subsection III (adverse reactions) | Narrative double-zero report; not estimable as a log RR | Both arms explicitly reported zero adverse reactions. |
| Si Daolin 2015 | Oxiracetam + nimodipine vs nimodipine | Reported adverse reactions | During the 14-day treatment | Oxiracetam + nimodipine; 0/62 | Nimodipine; 1/68 | PDF p.3, Methods 1.6; p.4, Results 2.4 | Narrative single-study safety evidence | The control arm reported one rash; this is not a double-zero report. |
| Sui 2019 | Oxiracetam add-on / balanced background vs control | Consciousness recovery | During the 12-day treatment | Oxiracetam + background therapy; 34/40 | Control/background therapy; 27/40 | PDF p.3, Results 2.2 | Narrative only | Definition differs from investigator-defined clinical response and was not pooled with that outcome. |
