# Supplementary material for: Efficacy and safety of oxiracetam for cognitive and functional outcomes after acute traumatic brain injury in Chinese populations: a systematic review and meta-analysis of randomized controlled trials
Source: Front Pharmacol. 2026 Sep 21;17:1894256. doi: 10.3389/fphar.2026.1894256 (PMC13635107; doi:10.3389/fphar.2026.1894256)
Supplement: Supplementary file 8 [file Table8.docx]

**Supplementary Table S7. Barthel Index measurement characteristics**

All five reports explicitly named the Barthel Index (BI), used a 0–100 scale, and scored higher values as better function. Exact versions and item wording were not reported; therefore, the pooled MD is interpreted as a summary difference in reported Barthel Index scores rather than as a uniform point gain in functional independence.

| **Study** | **Comparison** | **Instrument** | **Score range** | **Direction / construct** | **Assessment time** | **Analysis handling** | **Version and comparability** |
| --- | --- | --- | --- | --- | --- | --- | --- |
| Guo Xiaoming 2016 | Oxiracetam add-on / balanced background vs control | Barthel Index (BI) | 0-100 | Higher = better basic functional independence | End of treatment (2 weeks) | Included in the add-on/balanced-background Barthel Index MD synthesis | Exact version and item wording not reported; the named 0–100 Barthel Index construct was consistent, but timing differed and clinical interpretation remains cautious. |
| Guo Yongxiang 2015 | Oxiracetam add-on / balanced background vs control | Barthel Index (BI) | 0-100 | Higher = better basic functional independence | End of treatment (2 weeks) | Included in the add-on/balanced-background Barthel Index MD synthesis | Exact version and item wording not reported; the named 0–100 Barthel Index construct was consistent, but timing differed and clinical interpretation remains cautious. |
| Zhou 2013 | Oxiracetam add-on / balanced background vs control | Barthel Index (BI) | 0-100 | Higher = better basic functional independence | End of treatment (21 days) | Included in the add-on/balanced-background Barthel Index MD synthesis | Exact version and item wording not reported; the named 0–100 Barthel Index construct was consistent, but timing differed and clinical interpretation remains cautious. |
| Huang 2012 | Oxiracetam add-on / balanced background vs control | Barthel Index (BI) | 0-100 | Higher = better basic functional independence | Week 6 (week 3 also reported) | Included in the add-on/balanced-background Barthel Index MD synthesis | Exact version and item wording not reported; the named 0–100 Barthel Index construct was consistent, but timing differed and clinical interpretation remains cautious. |
| Liu Bin 2014 | Oxiracetam vs piracetam | Barthel Index (BI) | 0-100 | Higher = better basic functional independence | End of treatment (21 days) | Analyzed separately in the piracetam-controlled stratum; not pooled with add-on studies | Exact version and item wording not reported; the named 0–100 Barthel Index construct was consistent, but timing differed and clinical interpretation remains cautious. |
